# Interfacial chemistry governs nanoparticle self-sorting during biomimetic crystallization

**DOI:** 10.1038/s41467-026-75685-3

**Published:** 2026-08-04

**Authors:** Wenting Chen, Zhuodi Fan, Pei Liu, Xiaohong Hu, Yihao Yang, Qin Li, Meijiang Wang, Jingjing He, Wenjun Zhang, Steven P. Armes, Yin Ning

**Affiliations:** 1 State Key Laboratory of Bioactive Molecules and Druggability Assessment, College of Chemistry and Materials Science, Jinan University, Guangzhou, China; 2 Guangdong Provincial Key Laboratory of Supramolecular Coordination Chemistry, College of Chemistry and Materials Science, Jinan University, Guangzhou, China; 3 Guangdong Basic Research Center of Excellence for Natural Bioactive Molecules and Discovery of Innovative Drugs, College of Chemistry and Materials Science, Jinan University, Guangzhou, China; 4 College of Resources and Environment, Huazhong Agricultural University, Wuhan, China; 5 School of Mathematical and Physical Sciences, University of Sheffield, Brook Hill, Sheffield, UK

**Keywords:** Nanocomposites, Organic-inorganic nanostructures, Nanoparticles

## Abstract

Biominerals comprise composite crystals in which organic constituents are spatially organized within mineral matrices with exquisite precision, giving rise to hierarchically ordered architectures. Despite extensive efforts, achieving precise control over organic–inorganic interactions to construct biomimetic composite materials with programmable composition and spatial organization remains a major challenge. Here we show that diblock copolymer nanoparticles with distinct compositions and dimensions—resembling pseudo-proteins—undergo spontaneous self-sorting during occlusion within growing calcite crystals, yielding artificial biominerals in which two nanoparticle populations are selectively localized in distinct crystalline domains. Using in situ monitoring techniques and atomic force microscopy, we demonstrate that this self-sorting process arises from nanoparticle surface chemistry, which leads to differing polymer–mineral interfacial interactions. Moreover, the resulting composite crystals exhibit spatiotemporal release of the occluded species, highlighting the potential for advanced delivery systems. More broadly, self-sorting occlusion establishes a conceptual framework for understanding the spatial organization of organic components in biominerals and provides a versatile strategy for the rational design of next-generation biomimetic materials.

## Abstract (web summary)

Despite extensive efforts, achieving precise control over organic–inorganic interactions to construct biomimetic composite materials with programmable composition and spatial organization remains challenging. Here, the authors show that diblock copolymer nanoparticles undergo spontaneous self-sorting during occlusion within growing calcite crystals, yielding artificial biominerals with selective localization of the nanoparticles in distinct crystalline domains.

## Introduction

In natural biominerals such as bones, teeth or shells, inorganic minerals are not isolated entities but are intricately interwoven with biopolymers (e.g. peptides) to form hierarchical hybrid architectures^1–4^. A striking feature of these systems is that the organic component is spatially partitioned within or around the crystalline domains, rather than merely randomly distributed^5,6^. Such remarkable structures are believed to be indispensable for conferring exceptional stiffness, toughness, and damage tolerance^7^. Thus, mimicking nature to synthesize composite crystals with similar properties has been a hot research topic for a long time^8–12^. Indeed, a few striking examples of synthetic materials designed to rival or even surpass the performance of natural biominerals have been reported^13–15^, such as macroscopic artificial pearl-layer-like materials via a mesoscopic-scale approach combining ‘assembly and mineralization’^16^ and artificial biominerals, exhibiting texture and defect structures similar to those of biogenic calcite crystals, prepared by incorporating anionic block copolymer micelles into a calcite matrix^17^.

Notably, prior biomimetic approaches have typically focused on the interaction between single organic components and minerals because using multiple components complicates the identification of causal relationships^18,19^. In contrast, natural biomineralization is usually a much more complex process involving a diverse array of proteins, polysaccharides, lipids, and other organic components^20,21^. To date, multi-component biomimetic studies remain rare, no doubt because it is extremely difficult to distinguish between all the possible interactions over multiple length scales using current analytical techniques. Resolving these challenges would represent a conceptual breakthrough while establishing general principles for organic–inorganic interactions that could transform fields far beyond biomineralization^22,23^. Such advances would not only enhance our fundamental understanding of how living systems achieve nanoscale control over crystalline architectures but also provide essential design principles for the rational synthesis of bioinspired hybrid materials with tailored structure and function^24–26^.

In this study, we design nanoparticles with precise surface chemistries and dimensions that serve as tunable surrogates for biomacromolecules and introduce a new experimental model to elucidate the fundamental parameters governing nanoparticle partitioning and segregation during crystallization. Systematic modulation of chemical functionality, size, and morphology provides important insights into how certain functional groups govern the spatially precise self-sorting occlusion of nanoparticles within an inorganic crystalline matrix. Herein, self-sorting occlusion refers to the spontaneous and selective incorporation of two different guest nanoparticles within distinct regions of the host matrix. Our approach not only offers a useful model for understanding natural biomineralization mechanisms but also establishes a conceptual framework for the spatially controlled integration of organic and inorganic materials, with implications for the design of next-generation biomimetic hybrid materials.

## Results

### Synthesis and characterization of diblock copolymer nanoparticles

**Fig. 1:**
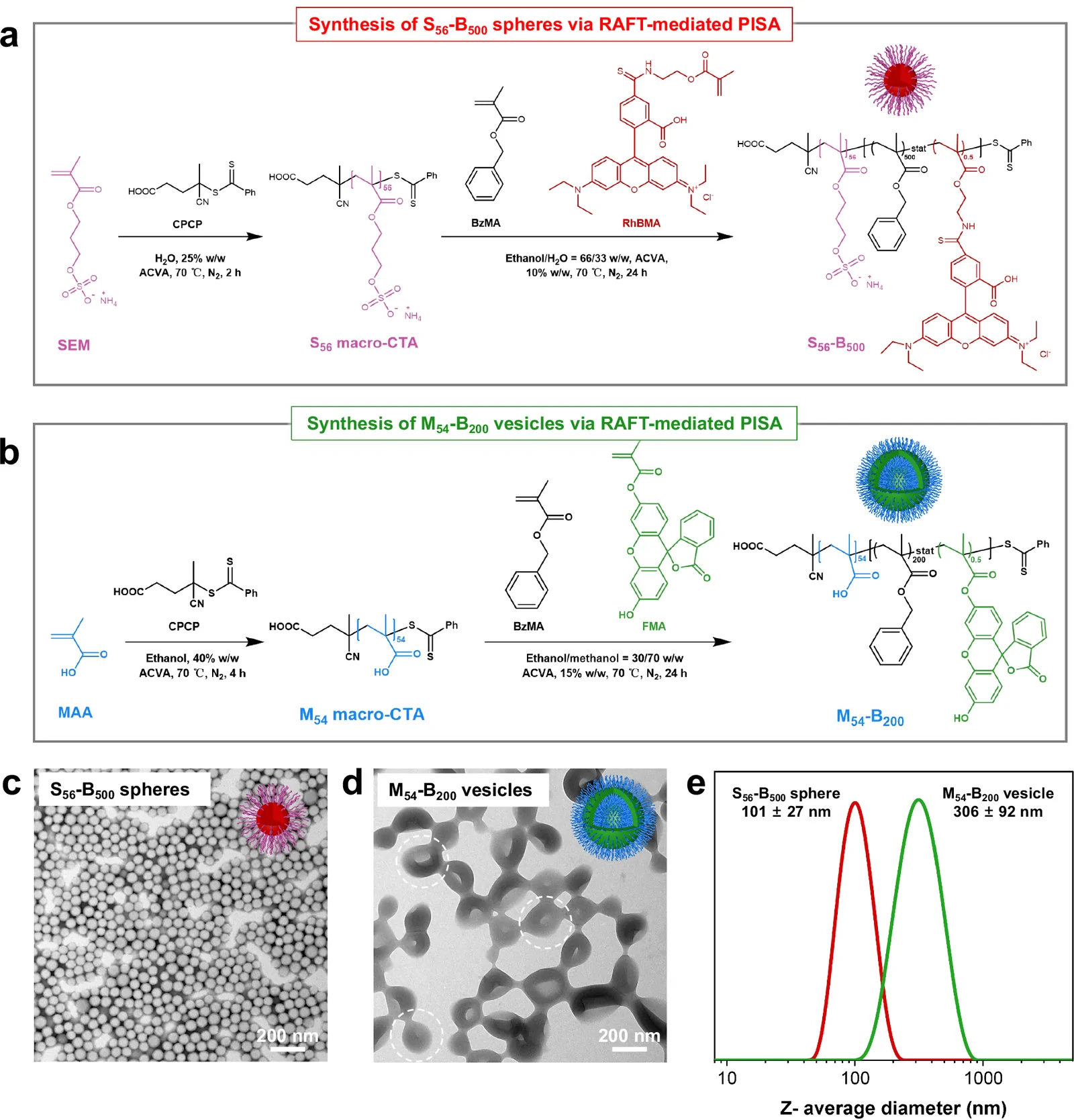
Controlled synthesis of diblock copolymer spheres or vesicles via RAFT-mediated PISA. **a** Synthetic protocol for the preparation of rhodamine-functionalized poly(ammonium 2-sulfatoethyl methacrylate)_56_-*block*-poly(benzyl methacrylate)_500_ [S_56_-B_500_] spheres; **b** Synthetic protocol for the preparation of fluorescein-functionalized poly(methacrylic acid)_54_-*block*-poly(benzyl methacrylate)_200_ [M_54_-B_200_] vesicles; **c** TEM image of S_56_-B_500_ spheres; **d** TEM image of M_54_-B_200_ vesicles. The dashed circles indicate individual vesicles. Their apparent aggregation is attributed to drying artifacts arising during TEM sample preparation; **e** Particle size distributions for S_56_-B_500_ spheres and M_54_-B_200_ vesicles obtained from dynamic light scattering (DLS). Cartoons in (**c**, **d**) (top right) illustrate the corresponding copolymer morphologies.

Reversible addition-fragmentation chain transfer (RAFT) polymerization was combined with polymerization-induced self-assembly (PISA) to prepare a series of diblock copolymer nanoparticles with either sulfate-rich or carboxylate-rich surface chemistry. Initially, poly(ammonium 2-sulfatoethyl methacrylate)_56_ and poly(methacrylic acid)_54_ macromolecular chain transfer agents (denoted as S_56_ macro-CTA and M_54_ macro-CTA, respectively; the subscripts denote the mean degree of polymerization in each case) were synthesized by RAFT solution polymerization (Fig. 1a, b and Supplementary Fig. 1; Supplementary Table 1). Subsequently, these precursors were chain-extended with benzyl methacrylate (B) to form near-monodisperse, sterically-stabilized poly(ammonium 2-sulfatoethyl methacrylate)_56_-*block*-poly(benzyl methacrylate)_500_ [denoted as S_56_-B_500_] spheres or poly(methacrylic acid)_54_-*block*-poly(benzyl methacrylate)_200_ [M_56_-B_200_] vesicles (Fig. 1c, d and Supplementary Table 2). To facilitate confocal laser scanning microscopy (CLSM) analysis, both types of nanoparticles were fluorescently labeled by incorporating a small amount of either 2-(methacryloyloxy)ethyl thiocarbamoyl rhodamine B (RhBMA) or fluorescein *O*-methacrylate (FMA) into the core-forming poly(benzyl methacrylate) block (Fig. 1a, b). It is worth emphasizing that these nanoparticles were designed to have distinctive morphologies (spheres vs. vesicles, Fig. 1c, d) and sizes (101 ± 27 nm vs. 306 ± 92 nm, Fig. 1e) to aid their differentiation by electron microscopy. On the other hand, the mean DPs of their steric stabilizer chains (56 vs. 54) were deliberately matched to minimize the known influence of chain length on their occlusion within host crystals^18^. Notably, hydrodynamic diameters determined by dynamic light scattering (DLS, Fig. 1e) were larger than the mean diameters estimated from transmission electron microscopy (TEM, Supplementary Fig. 2). This is because DLS measures the effective size of nanoparticles dispersed in aqueous media, which includes the solvated layer of steric stabilizer chains^27^.

### Self-sorting occlusion of S_56_-B_500_ spheres and M_54_-B_200_ vesicles within calcite

**Fig. 2:**
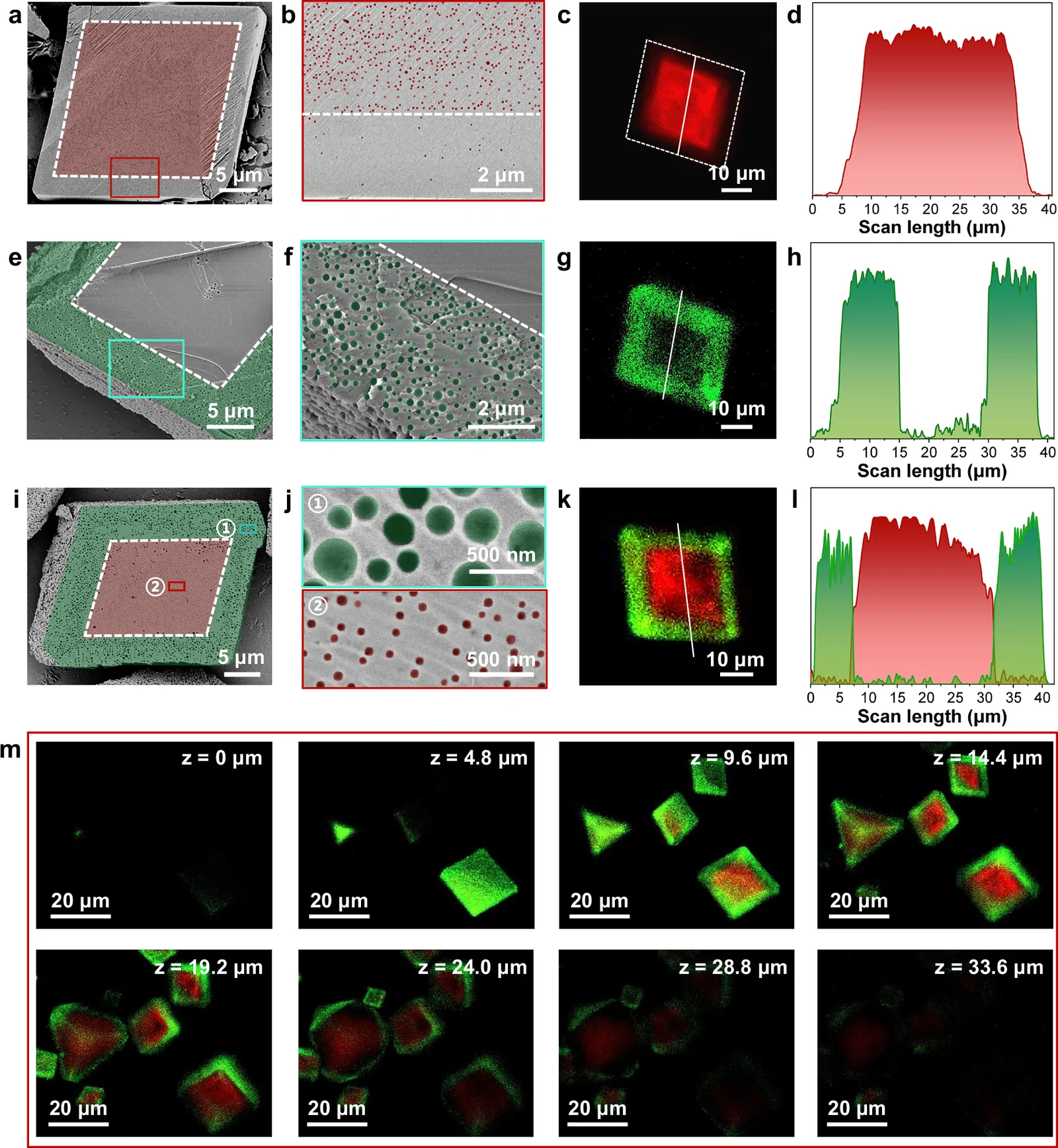
Self-sorting of S_56_-B_500_ spheres and M_54_-B_200_ vesicles within a calcite host crystal. These composite crystals were prepared in the presence of various additives: **a–d** S_56_-B_500_ spheres; **e–h** M_54_-B_200_ vesicles; **i-l**, a binary mixture of S_56_-B_500_ spheres and M_54_-B_200_ vesicles. **a, b, e, f, i, j**, Representative high-resolution SEM images of crystal cross-sections prepared by argon ion beam etching. For clarity, spheres and vesicles are pseudo-colored in red and green, respectively. **c, g, k** CLSM images of the composite crystals showing the spatial distribution of S_56_-B_500_ spheres (red) and M_54_-B_200_ vesicles (green). **d, h, l** Corresponding fluorescence intensity line-scan profiles. **m** Representative confocal fluorescence slices recorded at regular depths along the z-axis, revealing the localization of two distinct dye-labeled nanoparticles within a single composite crystal. Red fluorescence corresponds to RhBMA-labeled S_56_-B_500_ spheres, while green fluorescence denotes fluorescein-labeled M_54_-B_200_ vesicles.

As one of the most abundant minerals, calcite (CaCO_3_) was selected as a model host crystal^28^. In the absence of any additives, CaCO_3_ crystallizes to form transparent rhombohedra with smooth surfaces. In contrast, crystals grown in the presence of S_56_-B_500_ spheres or M_54_-B_200_ vesicles (or a binary mixture thereof) produced composite crystals with roughened surfaces (Supplementary Fig. 3). To examine their internal structure, argon ion beam etching was employed to expose cross-sections of the composite crystals, followed by field-emission scanning electron microscopy (SEM) analysis. This revealed distinctly different occlusion behavior. S_56_-B_500_ spheres were incorporated preferentially within the crystal cores, forming well-defined parallelogram-shaped domains aligned with the crystal facets (Fig. 2a, b). In contrast, M_54_-B_200_ vesicles were predominantly localized near the crystal surface (Fig. 2e, f). Remarkably, self-sorting occurred when both types of nanoparticles were present, such that the spheres and vesicles were selectively occluded within the central and surface regions, respectively (Fig. 2i, j). This indicates that the coexistence of S_56_-B_500_ spheres and M_54_-B_200_ vesicles does not interfere with their respective preferred occlusion behavior.

CLSM was used to examine the spatial distribution of the S_56_-B_500_ spheres and M_54_-B_200_ vesicles within the host crystals. CLSM images confirmed the core localization of S_56_-B_500_ spheres, the surface localization of M_54_-B_200_ vesicles, and their self-sorting behavior when employed as binary mixtures (Fig. 2c, d, g, h, k, l). Notably, fluorescence images recorded for slices of the host crystal provide compelling evidence for self-sorting: the two nanoparticle populations clearly occupy distinct regions (Fig. 2m and Supplementary Video 1). Importantly, both powder X-ray diffraction (XRD, Supplementary Fig. 4) and Raman spectroscopy (Supplementary Fig. 5) indicated that such nanoparticle occlusion does not alter the host crystal form, which is always calcite.

### In situ monitoring of the self-sorting occlusion of diblock copolymer nanoparticles

**Fig. 3:**
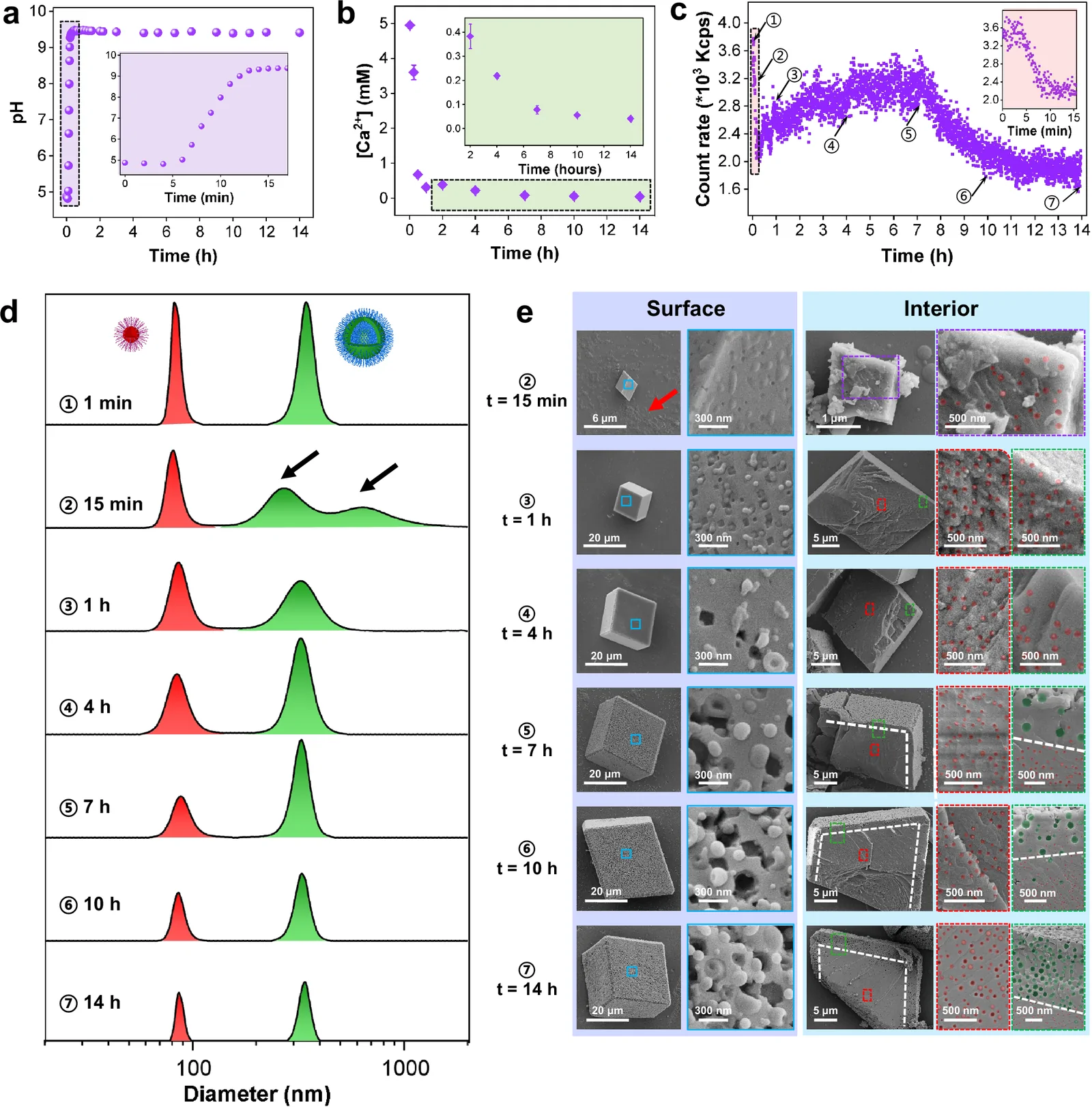
In situ monitoring of the self-sorting occlusion of S_56_-B_500_ spheres and M_54_-B_200_ vesicles during calcite crystal growth. **a** pH evolution during calcite crystallization; the inset highlights the initial 0–15 min period. **b** Variation of Ca^2+^ concentration as a function of crystallization time; the inset shows the 2–14 h interval. **c** DLS count rate as a function of time, with an inset showing the initial 0–15 min period. Variations in count rate reflect changes in nanoparticle concentration in solution during crystal growth. **d** Z-average hydrodynamic diameters were recorded at selected time points, where substantial shifts in the size distribution indicate subtle changes in colloidal stability. **e** SEM images of composite crystals recorded at the different time points, highlighting their surface morphology and internal structure. These data indicate the initial occlusion of S_56_-B_500_ spheres followed by the occlusion of M_54_-B_200_ vesicles over longer time scales, leading to their spatially segregated occlusion within calcite crystals via a self-sorting mechanism.

The composite crystals were precipitated using a well-established ammonia diffusion method (detailed procedure was provided in the Methods section)^29^. Upon initiation of crystallization, the solution pH increased rapidly (Fig. 3a), accompanied by a sharp decrease in the calcium ion concentration (Fig. 3b). The pronounced size disparity between the S_56_-B_500_ spheres and M_54_-B_200_ vesicles enables their clear differentiation by in situ DLS. Hence, this technique can be used to track the real-time evolution of both species during the growth of calcite crystals. In addition to size information, the scattered light intensity (or DLS count rate) is directly related to the nanoparticle concentration. As shown in Fig. 3c, the DLS count rate dropped sharply within the first 15 min, which is attributable to the aggregation of M_54_-B_200_ vesicles. This interpretation is supported by the intensity-weighted hydrodynamic size distribution recorded at *t* = 15 min, which exhibits two broad peaks (black arrows, Fig. 3d) that are characteristic of aggregation; vesicle aggregation is also corroborated by complementary SEM analysis (red arrow, Fig. 3e). Colloidal stability assays revealed that M_54_-B_200_ vesicles underwent flocculation when the Ca^2+^ concentration exceeded 3 mM, whereas S_56_-B_500_ spheres remained stable under the same conditions (Supplementary Fig. 6). Strikingly, the DLS count rate increased gradually between 15 min and 7 h (Fig. 3c), indicating spontaneous redispersion of the vesicle aggregates. This is attributed to the gradual depletion of Ca^2+^ from the aqueous reaction solution during calcite crystallization. At later times, the DLS count rate declined again and eventually reached a limiting value (Fig. 3c), with these observations being consistent with the occlusion of M_54_-B_200_ vesicles. Indeed, high-resolution SEM analysis of samples extracted at various time points revealed that spheres were continuously occluded from the onset up to approximately 4 h, after which vesicle occlusion commenced (Fig. 3e). Together, these observations provide clear evidence that spheres and vesicles undergo sequential self-sorting occlusion during calcite crystallization. Furthermore, the in situ DLS data indicate that the characteristic population corresponding to the S_56_-B_500_ spheres is always detected even during the latter stages of crystallization. This confirms that the S_56_-B_500_ spherical nanoparticles are still present in solution during occlusion of the M_54_-B_200_ vesicles within the growing crystals.

### Underlying mechanism for self-sorting occlusion

**Fig. 4:**
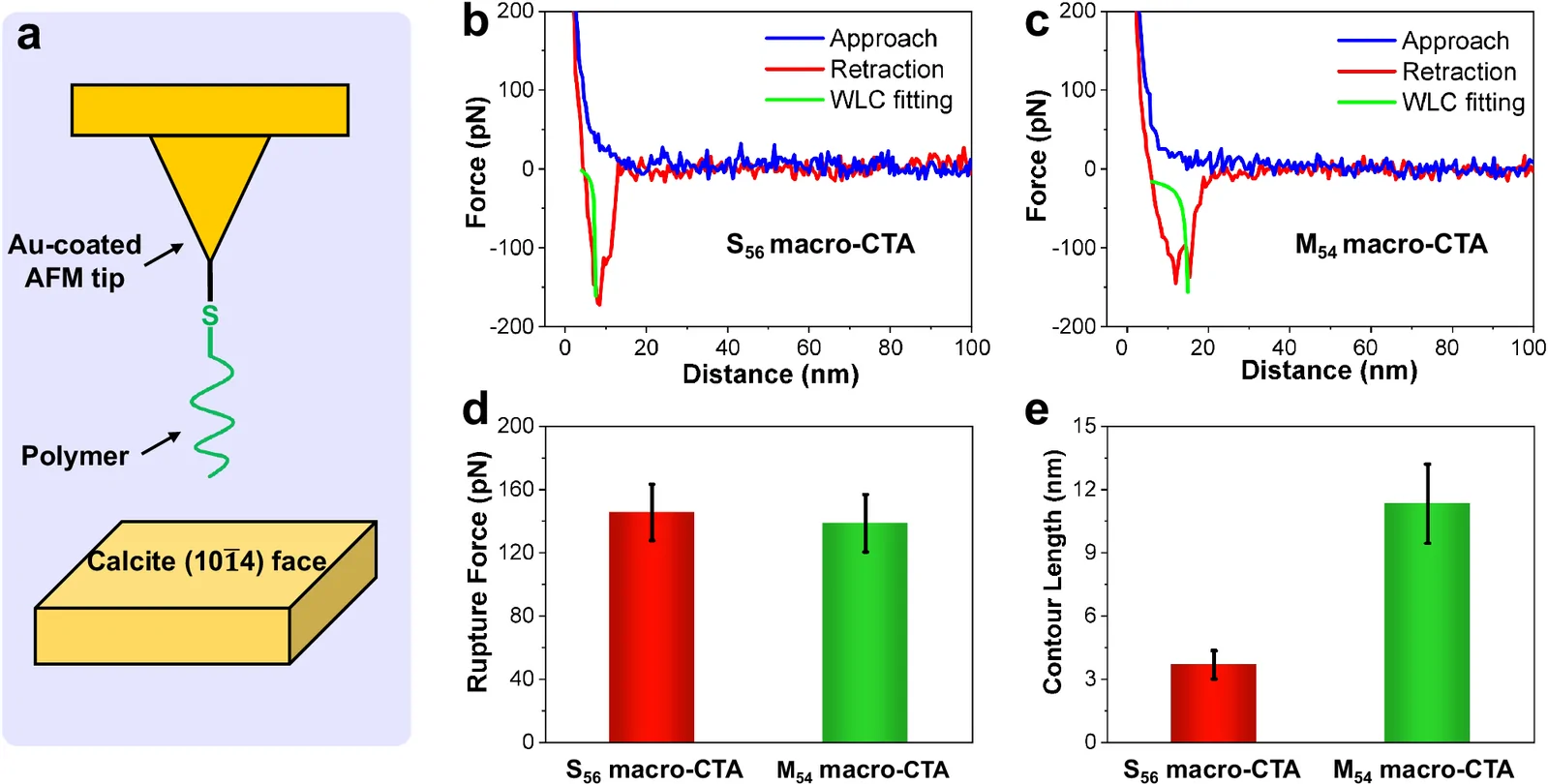
AFM-based force spectroscopy studies of polymer–calcite interactions in supersaturated solution (*σ* = 0.274). **a** Schematic representation of a force spectroscopy experiment, in which a single polymer chain (either M_54_ or S_56_) is tethered to an Au-modified AFM tip. Measurement of force–distance curves acquired with an AFM is used to probe the precise interaction between a single anionic steric stabilizer chain and the calcite surface. Representative force–distance curves showing the interaction of **b** S_56_ and **c** M_54_ with the surface of a growing calcite crystal. Statistical analysis of **d** rupture forces and **e** contour lengths for such polymer–calcite interactions. Error bars in (**d, e**) represent standard deviations calculated from ten independent measurements (*n* = 10).

Superficially, the self-sorting occlusion of S_56_-B_500_ spheres and M_54_-B_200_ vesicles is attributed to reversible aggregation/redispersion of the latter nanoparticles at high/low Ca^2+^ concentration. However, such behavior raises an interesting question: is the variable colloidal (in)stability of M_54_-B_200_ vesicles solely responsible, or is there a deeper molecular mechanism at play? Nanoparticle occlusion within inorganic crystals is closely related to their ability to interact intimately with the growing crystal surface^30,31^, with a strong surface interaction being essential for overcoming interfacial incompatibility and hence driving occlusion^32^. Both S_56_-B_500_ spheres and M_54_-B_200_ vesicles interact with calcite crystals through their respective polyelectrolytic stabilizer chains (S_56_ and M_54_). To probe these interactions, force spectroscopy experiments were performed using an atomic force microscope (AFM) to compare the interactions of both S_56_-B_500_ spheres and M_54_-B_200_ vesicles with the growing calcite ($10\bar{1}4$) crystal face (Fig. 4). First, individual steric stabilizer chains (i.e., S_56_ or M_54_) were tethered to Au-coated AFM tips (Fig. 4a) and then force-distance curves were recorded as the tip approached and retracted from the growing calcite surface. In each case, retraction curves were fitted using the well-known worm-like chain (WLC) model to extract rupture forces and molecular contour lengths (Fig. 4b, c and Supplementary Fig. 7)^33^. Rupture forces were comparable for both S_56_ and M_54_ (Fig. 4d), but the latter exhibited a longer contour length, indicating stronger binding to the calcite surface (Fig. 4e). Given their similar degrees of polymerization, these AFM studies indicate that anionic M_54_ chains interact more strongly with calcite than S_56_ chains during crystallization.

The underlying mechanism for self-sorting occlusion is clearly complex. Notably, further experiments confirmed that the relatively large S_30_-B_300_ vesicles (220 ± 73 nm diameter) were occluded within the central region of the calcite crystals (Supplementary Fig. 8), similar to the behavior observed for the relatively small S_56_-B_500_ spheres (101 ± 27 nm diameter). This observation indicates that the occlusion behavior of these sulfate-functionalized nanoparticles within calcite crystals is governed neither by the mean DP of their poly(ammonium 2-sulfatoethyl methacrylate) steric stabilizer chains nor by their particle size. Furthermore, the mean DP of the poly(methacrylic acid) chains also does not influence the occlusion behavior. Both M_36_-B_200_ (220 ± 38 nm) and M_85_-B_300_ (342 ± 99 nm) vesicles, which comprise shorter and longer steric stabilizer chains, respectively, are incorporated as a surface-confined layer within growing calcite crystals (Supplementary Fig. 9), which is consistent with the behavior observed for M_54_-B_200_ vesicles. Such experiments confirm that the occlusion behavior observed for the two types of nanoparticles is neither influenced by their morphology (i.e., spheres or vesicles) nor by the DP of the steric stabilizer block under the experimental conditions in this study. Furthermore, our previous work has shown that occlusion behavior remains unaffected even when the guest nanoparticles reach micrometer-scale dimensions or exhibit highly anisotropic morphologies (e.g., rod-like structures)^34,35^.

**Fig. 5:**
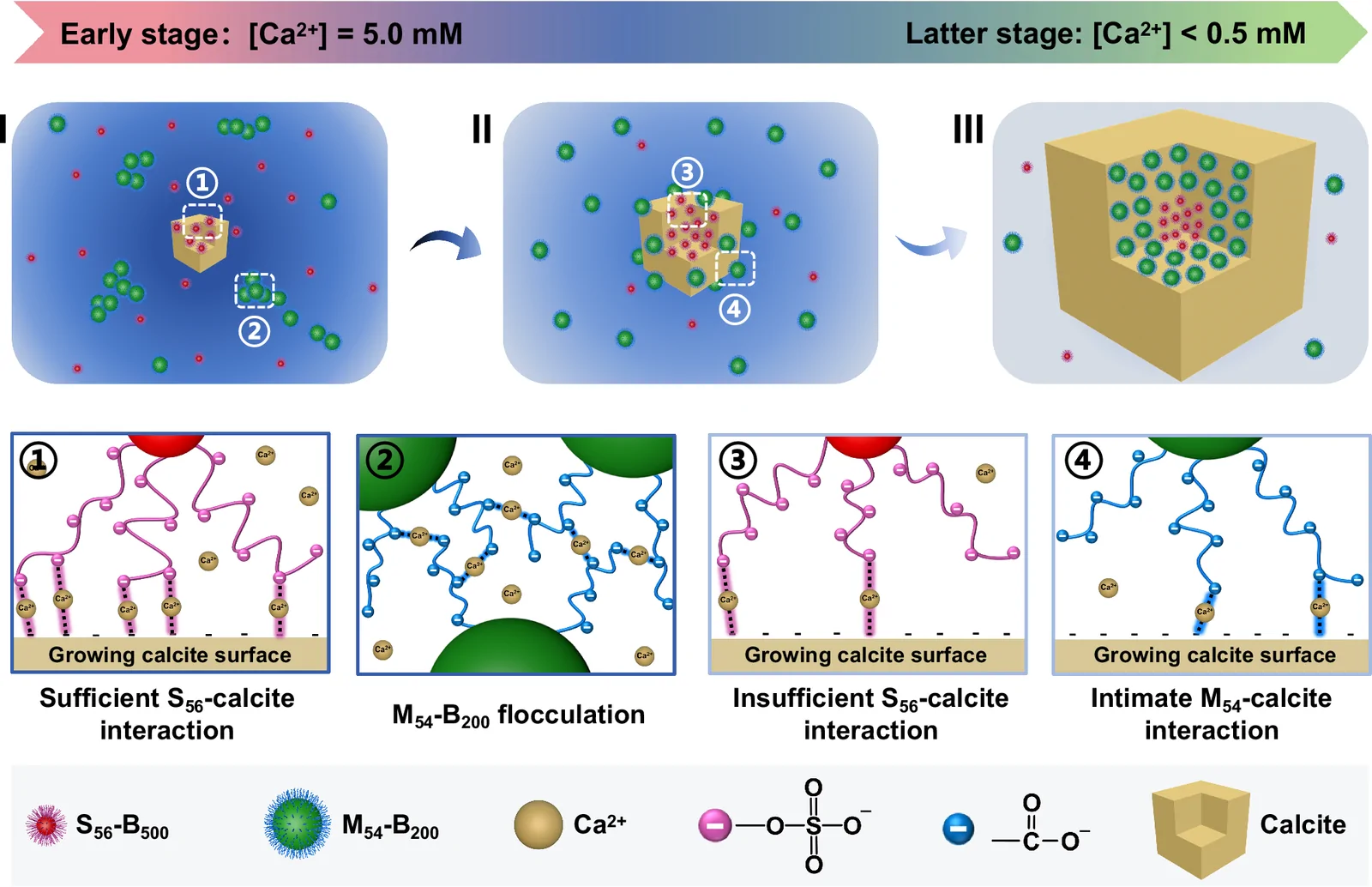
Schematic representation of the self-sorting occlusion mechanism for S_56_-B_500_ spheres and M_54_-B_200_ vesicles within calcite crystals. The progressive reduction in the solution concentration of Ca^2+^ during the formation of calcite crystals influences the occlusion behavior for these two types of nanoparticles. The high initial [Ca^2^⁺] ensures that only the colloidally stable S_56_-B_500_ spheres are occluded (mediated by sufficiently strong S_56_–calcite interactions under high [Ca^2^⁺]), whereas the M_54_-B_200_ vesicles undergo flocculation and are excluded. However, spontaneous redispersion of these aggregated M_54_-B_200_ vesicles occurs as the [Ca^2^⁺] is reduced, which enables their efficient occlusion (II). Notably, the occlusion of S_56_-B_500_ spheres terminates at lower [Ca^2^⁺], whereas that of M_54_-B_200_ vesicles persists, owing to the stronger interaction of M_54_ steric stabilizer chains with the calcite surface compared to S_56_ chains, as evidenced by AFM force–distance measurements. This difference gives rise to the self-sorting occlusion behavior (III).

The Ca^2+^ concentration is a critical parameter during nanoparticle occlusion. Initially, [Ca^2+^] = 5 mM, but as the calcite crystals are formed, the Ca^2+^ ions are progressively depleted from the aqueous reaction solution (Fig. 3b). The Ca^2+^ ions not only serve as a precursor for calcite formation, but also act as ionic bridge between the anionic nanoparticles and the growing calcite crystals, which facilitates nanoparticle occlusion within the host crystal (Fig. 5)^34,36^. Colloidal stability and interfacial interactions are both important, but their roles differ for the two types of nanoparticles and evolve over the course of crystallization. For S_56_-B_500_ spheres, the sulfate-based anionic stabilizer chains are strong polyelectrolytes with good salt tolerance, thus ensuring that these nanoparticles retain their colloidal stability throughout the entire process. This provides a critical condition for the occlusion of S_56_-B_500_ spheres (state $\textcircled{1}$, Fig. 5). However, during the latter stages of calcite growth, the Ca^2+^ concentration decreases substantially (e.g., below 1 mM), weakening the Ca^2+^-mediated ionic bridging (state $\textcircled{3}$, Fig. 5). Moreover, the intrinsic interaction between the S_56_ chains and the calcite surface is relatively weak compared with that of the M_54_ chains, as demonstrated by the AFM force measurements (Fig. 4e). Consequently, once the ionic bridging is diminished, S_56_-B_500_ spheres exhibit only limited affinity for the growing calcite surface and are no longer efficiently incorporated during subsequent crystal growth. As a result, S_56_-B_500_ spheres are predominantly retained within the crystal cores, having been occluded during the early stages of crystallization.

The situation is more complex for M_54_-B_200_ vesicles. At a relatively high Ca^2+^ concentration, M_54_-B_200_ vesicles form large aggregates and precipitate, therefore occlusion cannot occur (state $\textcircled{2}$, Fig. 5). However, after a substantial reduction in the Ca^2+^ concentration, these flocculated vesicles undergo spontaneous redispersal to enable the belated occlusion of individual M_54_-B_200_ vesicles (state $\textcircled{4}$, Fig. 5). This explains why M_54_-B_200_ vesicles are predominantly located within the near-surface layer of the calcite crystals. The occlusion of M_54_-B_200_ vesicles within calcite at such low Ca^2+^ concentrations is attributed to a stronger interaction between the M_54_ steric stabilizer chains and the calcite surface, as demonstrated by the force spectroscopy studies (Fig. 4). Indeed, when the initial Ca^2+^ concentration was reduced to 0.5 mM, M_54_-B_200_ vesicles could be efficiently occluded, whereas S_56_-B_500_ spheres were excluded from the calcite crystals (Supplementary Fig. 10). Interestingly, the occlusion behavior of either S_56_-B_500_ spheres or M_54_-B_200_ vesicles alone is the same as that observed for a binary mixture of these two species. In other words, the presence of one type of nanoparticle does not influence the occlusion behavior exhibited by the other. Remarkably, an overlap region was identified in which both spheres and vesicles underwent occlusion, particularly when using vesicles bearing a relatively short steric stabilizer block (e.g., M_36_-B_200_ vesicles, Supplementary Fig. 11).

### Universality study

To examine whether self-sorting occlusion is a generic phenomenon, we performed further experiments. Given that the occlusion behavior of guest nanoparticles is closely governed by their surface chemistry, we posited that if relatively large guest microparticles were coated with nanoparticles, then the occlusion behavior of the former system should reflect that of the latter. To examine this hypothesis, two new types of diblock copolymer nanoparticles were prepared via RAFT-mediated PISA: poly(potassium 3-sulfopropyl methacrylate)_57_-*block*-poly(styrene-*alt*-*N*-phenylmaleimide)_100_ [SK_57_-P(St-*alt*-NMI)_100_] spheres and poly(methacrylic acid)_75_-*block*-poly(styrene-*alt*-*N*-phenylmaleimide)_100_ [M_75_-P(St-*alt*-NMI)_100_] spheres (Supplementary Table 3 and Supplementary Fig. 12). Notably, the sulfonate-based steric stabilizer block was selected for its chemical similarity to the sulfate-based steric stabilizer reported above. Instead of employing SK_57_-P(St-*alt*-NMI)_100_ and M_75_-P(St-*alt*-NMI)_100_ directly for self-sorting occlusion experiments, we electrostatically adsorbed these two types of anionic nanoparticles onto cationic spherical mesoporous silica (MSNs-NH_2_) and octahedral zirconium-based metal-organic framework (UiO-66-NH_2_) particles respectively to form core**–**satellite composite microparticles (Supplementary Fig. 13). If the self-sorting occlusion mechanism is indeed dictated by surface chemistry, these composite particles should exhibit the occlusion behavior of either SK_57_-P(St-*alt*-NMI)_100_ or M_75_-P(St-*alt*-NMI)_100_ based on their respective surface compositions. As anticipated, neither the uncoated MSNs-NH_2_ nor the uncoated UiO-66-NH_2_ particles alone underwent occlusion within calcite crystals (Supplementary Fig. 14). However, SK_57_-P(St-alt-NMI)_100_-decorated MSNs-NH_2_ particles were incorporated within the cores of calcite crystals, whereas M_75_-P(St-*alt*-NMI)_100_-decorated UiO-66-NH_2_ particles were incorporated into the near-surface layers of calcite crystals (Supplementary Fig. 15a–h). Furthermore, using a binary mixture of these two types of microparticles led to self-sorting occlusion behavior (Supplementary Fig. 15i–l). These nanoparticles with varying sizes and morphologies exhibit consistent occluding behaviors due to their uniform chemical properties on the nanoparticle surfaces. These results further demonstrate that self-sorting occluding is primarily determined by the chemical characteristics of the nanoparticle surfaces, rather than by other factors.

## Discussion

There have been many reports on the effect of single-component additives such as soluble (bio)macromolecules or nanoparticles on crystal nucleation and growth^37–40^. The proliferation of such studies is no doubt related to their relative simplicity, which allows straightforward interpretation of causal relationships. In contrast, multi-component additive systems have been rarely studied. Herein, RAFT-mediated PISA provides a robust platform technology for the rational synthesis of S_56_-B_500_ spheres and M_54_-B_200_ vesicles with well-defined surface chemistry and morphology^41,42^. Such precise control is essential for elucidating the self-sorting occlusion mechanism, as it enables clear differentiation between the two types of nanoparticles when assessing their respective spatial distributions within calcite (Fig. 2). Moreover, it allows the use of in situ DLS to monitor the development of self-sorting occlusion during calcite growth (Fig. 3).

This study introduces a new concept that offers valuable physical insights into both natural biomineralization and the design of advanced materials. It is well-established that various organic molecules such as proteins and polysaccharides guide biomineralization by interacting selectively with crystal surfaces^43^. Similarly, nanoparticles bearing differing surface chemistry—sulfate-functionalized S_56_-B_500_ spheres and carboxylate-functionalized M_54_-B_200_ vesicles – exhibit distinctive occlusion behavior during calcite crystallization (Fig. 2). This self-sorting behavior closely resembles the selective incorporation of biomolecules in natural biominerals^6,44^, whereby specific organic components occupy well-defined crystal domains to achieve hierarchical organization and functional specialization^7,45^. Other forms of self-sorting phenomena have also been reported. For example, in poly(ε-caprolactone) (PCL) spherulites, the degree of crystallinity exhibits a spatial distribution along the radial direction, with lower crystallinity observed at the spherulite center. Consequently, the central region of such spherulites melts at a lower temperature than that of the outer region.^46^.

The ability to precisely control the spatial distribution of nanoparticles within an inorganic matrix opens up new avenues for designing artificial biominerals and advanced composite materials^47,48^. A formidable challenge when attempting to replicate natural biomineralization is achieving the selective incorporation of organic components into the mineral matrix. Self-sorting occlusion provides a promising strategy to address this daunting problem, since it potentially enables the formation of synthetic composite crystals with spatially graded distributions of functional groups or biomolecules. By tuning the surface chemistry of nanoparticles, their occlusion can be directed to specific regions of the matrix, leading to hierarchical materials with enhanced toughness, hardness and durability^15,49–51^.

**Fig. 6:**
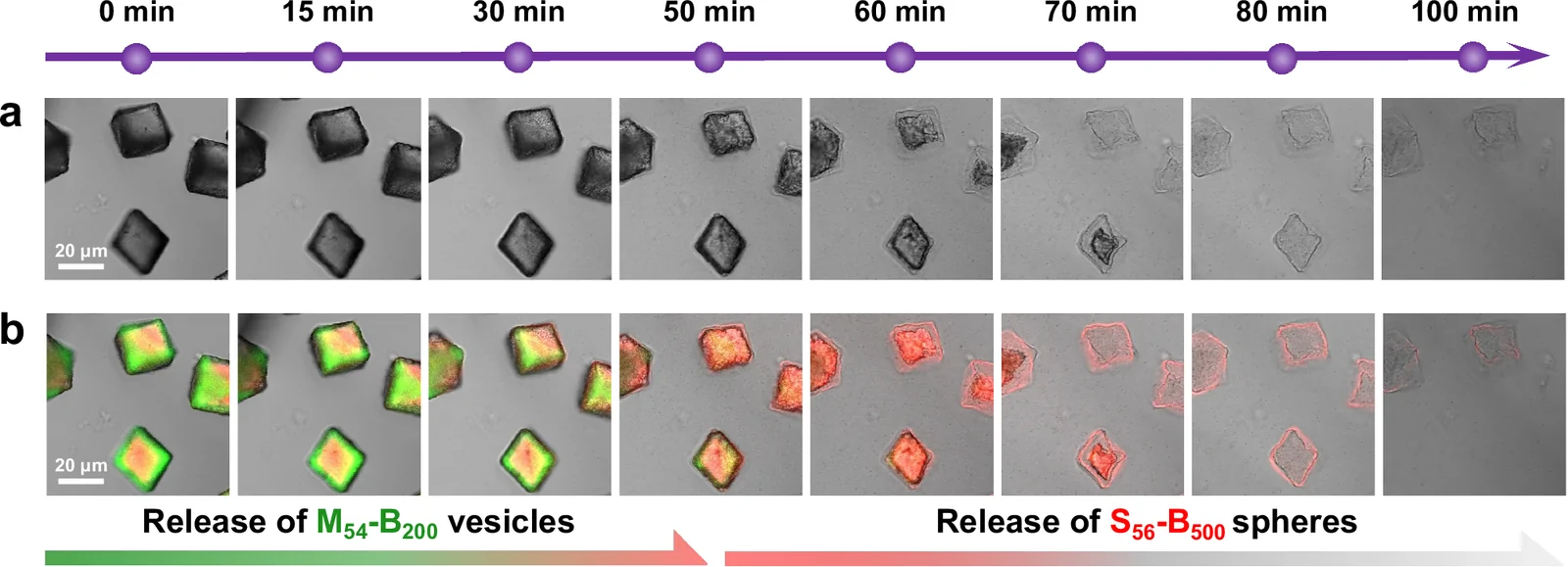
pH-triggered sequential release of vesicles and spheres monitored by CLSM in pH 4 buffer. **a** Bright-field images acquired at selected time points. **b** Corresponding CLSM images. The green fluorescence from M_54_-B_200_ vesicles progressively diminishes (0 → 50 min), followed by the gradual loss of red fluorescence from S_56_-B_500_ spheres (50 → 100 min), demonstrating the programmable, sequential release of two distinct species occluded at differing locations within the composite crystal.

Calcium carbonate is both biocompatible and pH-sensitive, making it an attractive carrier material for controlled delivery^52^. This prompts a key question: can spatiotemporal release be achieved by exploiting the exquisite control over the crystal structure provided by self-sorting occlusion? If acid dissolution of CaCO_3_ involves a surface etching mechanism, then nanoparticles located at or near the crystal surface should be released first, while those nanoparticles embedded deeper within the lattice should be liberated at a later stage. Such spatiotemporal release would offer a powerful design principle for multifunctional and smart delivery systems. To examine this concept, we monitored the acid-induced release of two types of fluorescently labeled nanoparticles embedded at distinct spatial locations within calcite crystals using real-time confocal microscopy at pH 4. Sequential release is clearly observed: green vesicles are liberated first, followed by the gradual release of red spherical nanoparticles (Fig. 6 and Supplementary Fig. 16). Hence, the self-sorting occlusion reported herein can be harnessed to achieve the programmed release of organic nanoparticles from calcite crystals. Given that such nanoparticles can be loaded with drugs or other actives^53^, this proof-of-concept experiment highlights the potential of this strategy for the development of spatiotemporal delivery applications.

In summary, diblock copolymer nanoparticles can undergo spatially segregated occlusion within calcite single crystals by a self-sorting mechanism. This transforms the traditional random incorporation of nanoparticles into a well-defined programmable process. This behavior is governed by the surface chemistry of the nanoparticles, which dictates their interactions with the growing crystal. Such precise control over nanoscale organization offers important insights for bio-inspired mineralization and suggests new opportunities for the rational design of multifunctional composite crystals, particularly in the context of potential delivery applications. Similar self-sorting mechanisms may also be relevant in the context of natural biomineralization, although verifying this hypothesis would no doubt require substantial further research.

## Methods

### Synthesis of poly(ammonium 2-sulfatoethyl methacrylate)_56_ macromolecular chain transfer agent (S_56_ macro-CTA)

A round-bottom flask equipped with a magnetic stirrer was charged with a 25% w/v aqueous solution of SEM monomer (120 g, 0.13 mol, target DP = 50). CPCP (0.74 g, 2.64 mmol) and the ACVA initiator (0.15 g, 528 μmol; [CPCP]/[ACVA] molar ratio = 5.0) were then added after adjusting the solution pH to 6 with 1 M NaOH. The flask was sealed with a rubber septum, and the reaction solution was purged with N₂ for 30 min before immersion in a preheated oil bath at 70 °C. Polymerization proceeded for 2 h and was quenched by cooling the flask in an ice/water bath and exposing the reaction mixture to air. The resulting polymer was dissolved in methanol and filtered to remove residual insoluble monomer; the filtrate was subsequently added dropwise into excess dichloromethane under continuous stirring to precipitate the polymer. The solvent was carefully decanted, and the precipitate was redissolved in water and freeze-dried overnight to yield the macro-CTA. A mean degree of polymerization (DP) of 56 was determined by ^1^H NMR spectroscopy, based on the integrated signal intensity of the methacrylic backbone relative to that of the aromatic protons. The synthesis of S_30_ macro-CTA was slightly modified by changing the target DP to 30. The synthetic procedure and GPC data are summarized in Supplementary Table 1.

### Synthesis of poly(methacrylic acid)_54_ macromolecular chain transfer agent (M_54_ macro-CTA)

A round-bottomed flask was charged with MAA (20.0 g, 232.4 mmol; target DP = 70), CPCP (0.93 g, 3.3 mmol), ACVA initiator (186 mg, 0.66 mmol; [CPCP]/[ACVA] molar ratio = 5.0), and ethanol (31.4 g). The flask was sealed, degassed under nitrogen for 30 min at 0 °C, and then immersed in a preheated oil bath at 70 °C for 3 h. The polymerization was quenched by cooling the reaction mixture in an ice bath and exposing it to air. The crude polymer was purified by three successive precipitations into a tenfold excess of diethyl ether. The purified macro-CTA was finally dissolved in water and lyophilized to afford a pink powder. The mean DP of the macro-CTA was determined by ^1^H NMR spectroscopy, based on the integrated signal intensity of the methacrylic backbone relative to that of the aromatic protons. The synthetic procedure and GPC data are summarized in Supplementary Table 1.

### Synthesis of poly(ammonium 2-sulfatoethyl methacrylate)_56_-*block*-poly(benzyl methacrylate)_500_ (S_56_-B_500_) block copolymer spheres labeled with rhodamine via RAFT dispersion polymerization

S_56_ macro-CTA (195 mg, 15 μmol), ACVA (0.84 mg, 3 μmol, [S_56_ macro-CTA]/[ACVA] molar ratio = 5.0), 1:2 w/w water/ethanol (13.6 g), RhBMA (4.9 mg, 7.5 μmol) and benzyl methacrylate monomer (1.322 g, 7.5 mmol, target DP = 500) were added to a reaction flask. The flask was then sealed, purged with nitrogen for 30 min, and immersed in a preheated oil bath at 70 °C for 24 h. The copolymerization was terminated by cooling the reaction flask using an ice bath and exposing the reaction mixture to air. The resulting S_56_-B_500_ block copolymer spheres were purified by dialysis against deionized water for 3 days using dialysis tubing with a molecular weight cut-off of 25,000 Da.

### Synthesis of poly(methacrylic acid)_x_-*block*-poly(benzyl methacrylate)_y_ (M_x_-B_y_) block copolymer vesicles labeled with fluorescein via RAFT dispersion polymerization

A representative protocol for the synthesis of M_54_-B_200_ vesicles was as follows. M_54_ macro-CTA (99.7 mg, 20 μmol), ACVA initiator (1.1 mg, 4 μmol; [M_54_ macro-CTA] /[ACVA] molar ratio = 5.0), fluorescein methacrylate (FMA, 4.0 mg, 10 μmol), and 70:30 w/w methanol/ethanol (4.6 g) were added to a 10 mL glass vial equipped with a magnetic stir bar. Benzyl methacrylate (704.8 mg, 4.0 mmol; target DP = 200) was subsequently added to afford a 15% w/w solution. The vial was sealed and degassed under nitrogen for 30 min at 0 °C, then immersed in an oil bath at 70 °C for 24 h. ^1^H NMR analysis confirmed a final monomer conversion of more than 99%. The resulting M_54_-B_200_ vesicles were diluted to 0.2% w/w and transferred into water by five centrifugation–redispersion cycles (15 min at 9500 rpm per cycle). Synthetic protocols for the other diblock copolymer nanoparticles are summarized in Supplementary Table 2.

### Precipitation of calcium carbonate crystals in the presence of various block copolymer additives

Calcium carbonate crystals were precipitated using the ammonia diffusion method^29^. A Petri dish containing 10 mL of a 5 mM CaCl_2_ aqueous solution plus 0.05% w/w copolymer nanoparticles was placed in a desiccator. Glass slides (pre-cleaned using piranha solution) were positioned at the base of the Petri dish. Crystallization was induced by exposure to ammonium carbonate (2 g, placed at the bottom of the desiccator) for 24 h at 20 °C. Ammonium carbonate decomposes slowly to generate CO_2_ and NH_3_, which diffuse across the gas–liquid interface into the solution. Dissolved CO_2_ reacts with water to form carbonic acid, which subsequently forms carbonate and bicarbonate ions, whose relative concentrations are governed by the solution pH. Concurrently, dissolved NH_3_ increases the solution pH. In the presence of Ca^2+^ ions, these processes produce a solution that is supersaturated with respect to CaCO_3_. After crystallization, the glass slides were removed, washed three times with deionized water, and rinsed three times with ethanol. Each occlusion experiment was repeated at least twice, with consistent results being obtained.

## Supplementary information


Supplementary Information
Description of Additional Supplementary Files
Supplementary Video 1
Transparent Peer Review file


## Source data


Source Data


## Data Availability

The data that support the findings of this study are available in the paper and its Supplementary Information or from the corresponding author upon request. Source data are provided with this paper.
