# Supplementary material for: Interfacial chemistry governs nanoparticle self-sorting during biomimetic crystallization
Source: Nat Commun. 2026 Aug 4;17:9382. doi: 10.1038/s41467-026-75685-3 (PMC13534651; doi:10.1038/s41467-026-75685-3)
Supplement: Supplementary file 1 [file 41467_2026_75685_MOESM2_ESM.pdf]

Description of Additional Supplementary Files

File Name: Supplementary Video 1

Description: The video demonstrates that the two fluorescently labelled nanoparticles occupy distinct regions, providing direct evidence for self-sorting during crystal growth.
