# Supplementary material for: Interfacial chemistry governs nanoparticle self-sorting during biomimetic crystallization
Source: Nat Commun. 2026 Aug 4;17:9382. doi: 10.1038/s41467-026-75685-3 (PMC13534651; doi:10.1038/s41467-026-75685-3)
Supplement: Supplementary file 2 [file 41467_2026_75685_MOESM4_ESM.pdf]

nature portfolio

Peer Review File

Interfacial chemistry governs nanoparticle self-sorting during biomimetic crystallization

Corresponding Author: Professor Yin Ning

This file contains all reviewer reports in order by version, followed by all author rebuttals in order by version.

Version 0:

Reviewer comments:

Reviewer #1

(Remarks to the Author)
This manuscript by Chen et al. reports a highly interdisciplinary and carefully designed study on the self-sorting occlusion of diblock copolymer nanoparticles during calcite crystallization. By employing RAFT-mediated PISA to produce nanoparticles with well-defined and tunable surface chemistry, the authors demonstrate that two distinct nanoparticle populations can undergo spatially segregated incorporation within a single crystal via a self-sorting mechanism. The combination of in situ DLS monitoring, AFM force spectroscopy, and correlative microscopy provides a convincing mechanistic picture linking interfacial chemistry to macroscopic spatial organization.
Overall, this is a well-executed and conceptually significant study that advances our understanding of multi-component biomimetic mineralization. The work is particularly noteworthy because it moves beyond the widely studied single-additive systems and establishes a tractable experimental model for multi-component self-organization, which is highly relevant to natural biomineralization. I believe this manuscript is suitable for publication after minor revision. My specific comments are as follows:
1, The authors attribute the self-sorting behavior to a combination of (i) Ca^{2+}-dependent colloidal stability and (ii) differences in polymer–calcite interfacial interactions. This interpretation is convincing, but the relative roles of these two factors could be articulated more clearly. In particular, it would be helpful if the authors could briefly clarify whether the observed spatial segregation should be viewed primarily as a kinetically regulated process (i.e., sequential accessibility due to aggregation/redispersion), or whether interfacial binding strength plays a more decisive role in determining the final localization.
2, The manuscript convincingly emphasizes surface chemistry as the governing parameter. At the same time, the primary systems studied differ in both size and morphology (spheres vs. vesicles). Although control experiments are provided, it would be helpful to include a brief statement clarifying to what extent size/morphology effects can be considered secondary in the present system.
3, The phrase “with resemble properties” should be corrected to “with similar properties”?
4, The term “self-sorting occlusion” is well chosen, but it would benefit from a concise definition when first introduced.
5, The interpretation of count rate as a proxy for nanoparticle concentration is reasonable but indirect; a brief clarification of assumptions would be helpful.
6, In Figure 3, the correspondence between DLS signals and SEM snapshots could be highlighted more explicitly.
7, Figure 5 is clear but could be improved by explicitly labeling Ca^{2+} concentration regimes.

Reviewer #2

(Remarks to the Author)
This is an interesting paper showing nanoparticle self-sorting during its incorporation into a growing crystal.
It has been shown widely that the crystals as hosts will incorporate the surroundings as guests and become composites. And many groups including the authors are working for this topic for long. Nevertheless, this kind of incorporation is still surprising. This work shows new surprise that 2 types of polymer nanoparticles exhibit self-sorting during its incorporation into a growing crystal. Mechanisms for the self-sorting have been discussed in association with the varied interfacial chemistry. I will suggest to accept this paper after minor revisions.
1. Self-sorting of polymers like molecular weight fractionation is quite well known (e.g., European Polymer Journal 107 (2018) 303–307). This work shows one type of it although the fractionation mechanism is quite different.

2. The self-sorting mechanism is associated with the varied interfacial chemistry. It might deserve to examine thermodynamic driving force (like varied solubility of the two nanoparticles) and the kinetic possibility (like varied sizes of the two nanoparticles). For the kinetics, it was described (e.g., Chernov, A. A. Modern Crystallography III 1984) that large particles get incorporated easier.
3. The authors describe that 'M54-B200 vesicles form large aggregates that cannot undergo occlusion'. It is not explained why aggregate cannot get incorporated. Actually, aggregates should get incorporated even easier according to refs (e.g., Chernov, A. A. Modern Crystallography III 1984).
4. Language issues like 'syntheses'.

Reviewer #3

(Remarks to the Author)
The manuscript reports a new phenomenon involving particle occlusion within growing single crystals from a team with extensive expertise in this field. The results demonstrate that two distinct foreign particles can be incorporated into different regions of growing crystals, providing valuable insights into biomineralization and bio-inspired material synthesis. The findings are interesting and significant; however, I have several comments regarding the proposed mechanisms and interpretations.
1. For the TEM images shown in Fig. 1b, could the authors label one or two representative vesicles? Currently, it appears that many vesicles are cross-linked or aggregated together, making the structures difficult to distinguish.
2. More experimental details regarding the ammonia diffusion-controlled calcite growth should be provided in the SI.
3. In the co-incorporation experiments, were the spheres already depleted from solution when the vesicles began to incorporate into the crystals?
4. For the AFM force measurements, it is important to consider the loading rate (tip velocity). As the loading rate increases, the rupture force transitions from an equilibrium regime, which reflects bond/unbond events, to a kinetic regime where the measured force increases with loading rate. Therefore, the measurements should ideally be performed within the equilibrium regime to accurately determine binding energies. I assume the authors have considered this appropriately, but the relevant experimental details should be included in the SI. It is also suggested to present more representative force curves in SI.
5. On page 11, the authors introduce three additional particles (e.g., S30B300 and M36B200). How do the hydrodynamic diameters of these particles compare with those of the original systems? In addition, a transition sentence introducing this new set of experiments would improve the readability of the paragraph.
6. Assuming that the carbonate concentration remains relatively constant due to equilibration with atmospheric CO_2, the decrease in calcium concentration during growth would reduce the solution supersaturation and consequently decrease the calcite growth rate (e.g., step velocity). This introduces an important kinetic factor that should be considered in the discussion.
7. How do the relative bulk (and interfacial) diffusivities of the two particles compare? Since the vesicles are much larger and also appear prone to aggregation/cross-linking, one would expect them to possess substantially lower diffusivity. In this context, the thermodynamic parameters measured by AFM may not fully explain the observed behavior. Considering the simultaneous decrease in crystal growth kinetics, it may be possible to interpret the self-sorting phenomenon from a kinetic-control perspective: the smaller spheres, with faster diffusion and/or lower kinetic barriers, preferentially incorporate during initial periods of higher growth rate, whereas the larger vesicles incorporate later under slower growth conditions.
8. I am somewhat confused by the new experiments introduced immediately before the Discussion section involving microparticles. The results are interesting and appear to show some degrees of similar self-sorting behavior. However, because the spheres are already attached to the microparticles, would they still undergo the same aggregation–dispersion processes discussed previously? If not, the earlier explanation based on the colloidal stability of vesicles appears somewhat contradictory and may require clarification.
9. The authors mainly attribute the self-sorting behavior to changes in colloidal stability arising from variations in calcium concentration. One possible way to directly test this hypothesis would be to use a flow-cell setup to maintain a constant calcium concentration during crystal growth. This is only a suggestion and not intended as a required experiment, but it may help strengthen the mechanistic interpretation.

Version 1:

Reviewer comments:

Reviewer #1

(Remarks to the Author)
The authors have fully addressed all comments. I recommend the publication of this manuscript in its current form.

Reviewer #2

(Remarks to the Author)
The authors have addressed the issues well.

Reviewer #3

(Remarks to the Author)

The authors have well addressed my comments and have thoroughly revised the manuscript. The new data and text now clearly support the conclusion that the proposed self-sorting phenomenon is governed by interfacial chemistry rather than particle size or topology. I have one remaining minor comment; otherwise, I believe the manuscript is suitable for publication. The force spectroscopy was performed at low supersaturation, corresponding to the low-Ca-concentration regime rather than the initial high-Ca regime. The zeta potential data in Figure S6 show that the potential decreases with increasing Ca concentration, becoming less negative for both types of particles. Could the authors clarify why the interaction between S56 chains and the calcite surface increases with increasing Ca concentration? Is this enhancement primarily due to changes in the zeta potential of the calcite surface (and the particle), or does it reflect differences in chemical bonding strength?

Open Access This Peer Review File is licensed under a Creative Commons Attribution 4.0 International License, which permits use, sharing, adaptation, distribution and reproduction in any medium or format, as long as you give appropriate credit to the original author(s) and the source, provide a link to the Creative Commons license, and indicate if changes were made.

In cases where reviewers are anonymous, credit should be given to 'Anonymous Referee' and the source.

The images or other third party material in this Peer Review File are included in the article's Creative Commons license, unless indicated otherwise in a credit line to the material. If material is not included in the article's Creative Commons license and your intended use is not permitted by statutory regulation or exceeds the permitted use, you will need to obtain permission directly from the copyright holder.

To view a copy of this license, visit https://creativecommons.org/licenses/by/4.0/

Reviewer 1:

This manuscript by Chen et al. reports a highly interdisciplinary and carefully designed study on the self-sorting occlusion of diblock copolymer nanoparticles during calcite crystallization. By employing RAFT-mediated PISA to produce nanoparticles with well-defined and tunable surface chemistry, the authors demonstrate that two distinct nanoparticle populations can undergo spatially segregated incorporation within a single crystal via a self-sorting mechanism. The combination of in situ DLS monitoring, AFM force spectroscopy, and correlative microscopy provides a convincing mechanistic picture linking interfacial chemistry to macroscopic spatial organization. Overall, this is a well-executed and conceptually significant study that advances our understanding of multi-component biomimetic mineralization. The work is particularly noteworthy because it moves beyond the widely studied single-additive systems and establishes a tractable experimental model for multi-component self-organization, which is highly relevant to natural biomineralization. I believe this manuscript is suitable for publication after minor revision.

Reply: We thank the reviewer for these positive and encouraging comments. We are pleased that the reviewer recognizes the conceptual significance of our multi-component self-sorting strategy and the strength of the combined experimental approaches.

1. The authors attribute the self-sorting behavior to a combination of (i) Ca^{2+}-dependent colloidal stability and (ii) differences in polymer–calcite interfacial interactions. This interpretation is convincing, but the relative roles of these two factors could be articulated more clearly. In particular, it would be helpful if the authors could briefly clarify whether the observed spatial segregation should be viewed primarily as a kinetically regulated process (i.e., sequential accessibility due to aggregation/redispersion), or whether interfacial binding strength plays a more decisive role in determining the final localization.

Reply: We thank the reviewer for these insightful comments. In response, we would like to clarify that both colloidal stability and interfacial interactions are important. However, their relative contributions differ for the two nanoparticle populations and evolve over the course of crystallization. For the M_{54}-B_{200} vesicles, their poor colloidal stability at the initial high Ca^{2+} concentration leads to their aggregation (see Supplementary Fig. 6 and red arrow in Fig 3e), which prevents their effective occlusion during the early stages of crystallization. As crystallization continues, the Ca^{2+} concentration is reduced and the flocculated vesicles redisperse, enabling their belated incorporation into the growing calcite crystals (Fig 3e). During this stage, the strong interaction between anionic carboxylate groups and calcite enables M_{54}-B_{200} vesicles to be incorporated even at relatively low Ca^{2+} concentrations, which correspond to slower crystal growth. In contrast, S_{56}-B_{500} spheres remain colloidally stable throughout the entire crystallization process under the same conditions, allowing them to be incorporated from the beginning (Fig 3e). However, their occlusion efficiency diminishes significantly in the latter stages owing to the relatively low Ca^{2+} concentration. Under such conditions, the interaction between the S_{56} chains and the surface of the growing calcite crystals is no longer sufficient to ensure occlusion.

Accordingly, these points have been emphasized and clarified in the revised manuscript on pages 11-13, which now reads as follows: "*Colloidal stability and interfacial interactions are both important, but their roles differ for the two types of nanoparticles and evolve over the course of crystallization. For S_{56}-B_{500} spheres, the sulfate-based anionic stabilizer chains are strong polyelectrolytes with good salt tolerance, thus ensuring that these nanoparticles*

retain their colloidal stability throughout the entire process. This provides a critical condition for the occlusion of S_{56}-B_{500} spheres (state ①, Fig. 5). However, in the latter stages of calcite formation, the Ca^{2+} concentration is relatively low (e.g. below 1 mM) so the interaction between the S_{56} chains and the calcite surface is no longer sufficient to sustain occlusion (state ③, Fig. 5). This explains why S_{56}-B_{500} spheres are primarily incorporated within the cores of the calcite crystals. The situation is more complex for M_{54}-B_{200} vesicles. At a relatively high Ca^{2+} concentration, M_{54}-B_{200} vesicles form large aggregates and precipitate, therefore occlusion cannot occur (state ②, Fig. 5). However, after a substantial reduction in the Ca^{2+} concentration, these flocculated vesicles undergo spontaneous redispersal to enable the belated occlusion of individual M_{54}-B_{200} vesicles (state ④, Fig. 5). This explains why M_{54}-B_{200} vesicles are predominantly located within the near-surface layer of the calcite crystals. The occlusion of M_{54}-B_{200} vesicles within calcite at such low Ca^{2+} concentrations is attributed to a stronger interaction between the M_{54} steric stabilizer chains and the calcite surface, as demonstrated by the force spectroscopy studies (Fig. 4). Indeed, when the initial Ca^{2+} concentration was reduced to 0.5 mM, M_{54}-B_{200} vesicles could be efficiently occluded, whereas S_{56}-B_{500} spheres were excluded from the calcite crystals (Supplementary Fig. 10).".

2. The manuscript convincingly emphasizes surface chemistry as the governing parameter. At the same time, the primary systems studied differ in both size and morphology (spheres vs. vesicles). Although control experiments are provided, it would be helpful to include a brief statement clarifying to what extent size/morphology effects can be considered secondary in the present system.

Reply: We thank the reviewer for this insightful comment. According to the calcite occlusion literature, as well as the findings of the present study, we note that the occlusion behavior of guest nanoparticles is largely insensitive to variation in their size and morphology. More specifically, our prior studies confirm that occlusion occurs even for micrometer-sized spherical or rod-like guest particles (see *Chem. Sci.*, 2019, *10*, 8964-8972; *Angew. Chem. Int. Ed.*, 2024, *63*, e202410908).

Accordingly, we now clarify this important point on page 11 of our updated manuscript, where the revised text reads as follows: "*Furthermore, our previous work has shown that occlusion behavior remains unaffected even when the guest nanoparticles reach micrometer-scale dimensions or exhibit highly anisotropic morphologies (e.g., rod-like structures)*[34,35]".

3. The phrase "with resemble properties" should be corrected to "with similar properties"?

Reply: The phrase has been corrected to "with similar properties" in the revised manuscript (see page 3).

4. The term "self-sorting occlusion" is well chosen, but it would benefit from a concise definition when first introduced.

Reply: We thank the reviewer for this constructive suggestion. Accordingly, a concise definition of self-sorting occlusion is now provided on page 4 of the updated manuscript: "*Herein, self-sorting occlusion refers to the spontaneous and selective incorporation of two different guest nanoparticles within distinct regions of the host matrix.*"

5. The interpretation of count rate as a proxy for nanoparticle concentration is reasonable but indirect; a brief clarification of assumptions would be helpful.

Reply: We thank the reviewer for this helpful comment. In our updated manuscript, a brief clarification has been included on page 8 to explicitly state the underlying assumption, which reads as follows: "*In addition to size information, the scattered light intensity (or DLS count rate) is directly related to the nanoparticle concentration.*"

6. In Figure 3, the correspondence between DLS signals and SEM snapshots could be highlighted more explicitly.

Reply: We thank the reviewer for this constructive suggestion. In the updated Fig. 3 (see page 9), the SEM images have been relabeled and more explicitly annotated to clarify their correspondence with the DLS signals. As a result, the relationship between these two datasets is now more explicit and hence readily understood by the reader.

7. Figure 5 is clear but could be improved by explicitly labeling Ca^{2+} concentration regimes.

Reply: This is a useful comment: both the initial and latter Ca^{2+} concentration regimes are now labeled in the updated Fig. 5, thereby improving its clarity (see below).

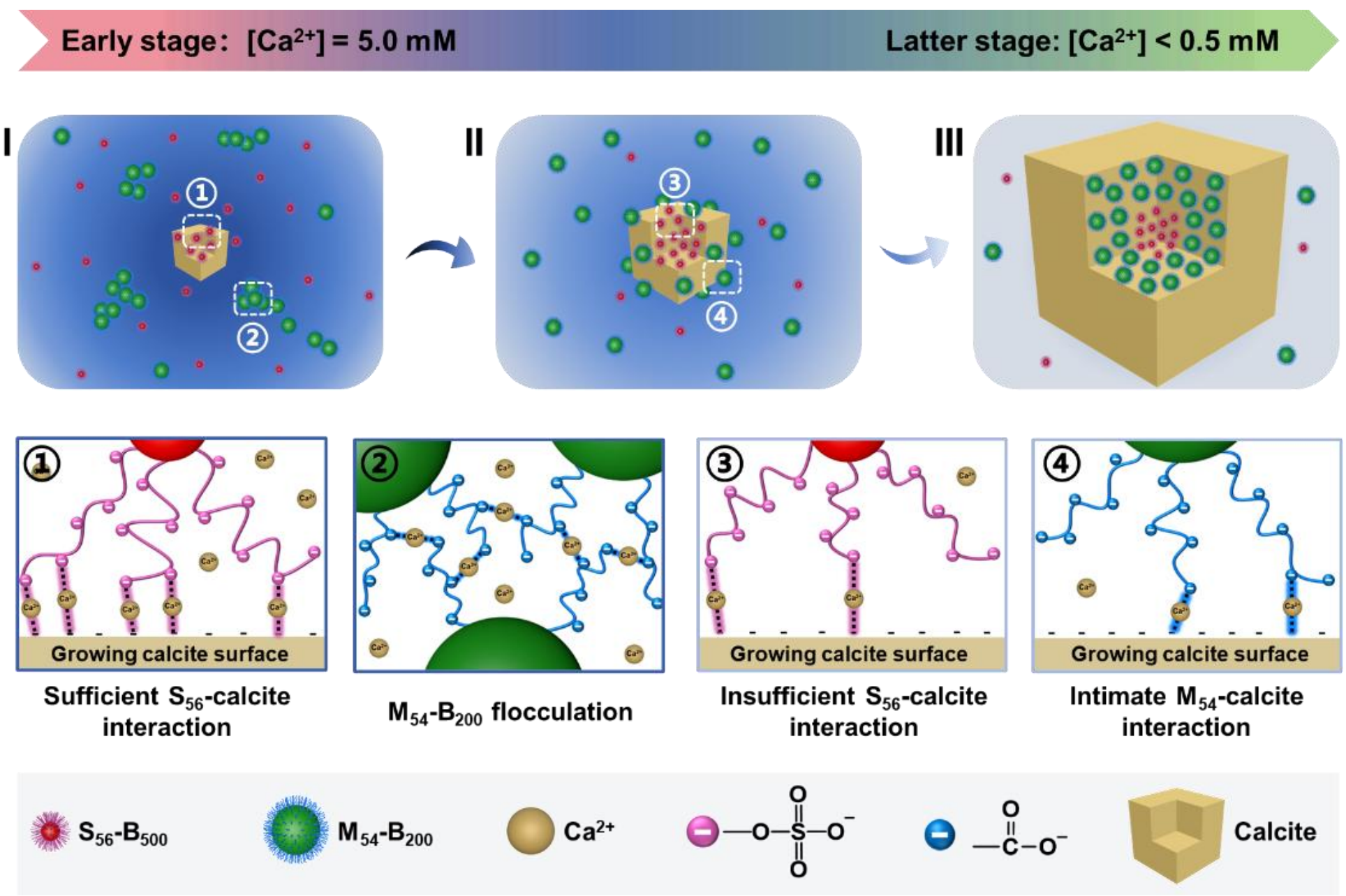

Reviewer 2:

This is an interesting paper showing nanoparticle self-sorting during its incorporation into a growing crystal. It has been shown widely that the crystals as hosts will incorporate the surroundings as guests and become composites. And many groups including the authors are working for this topic for long. Nevertheless, this kind of incorporation is still surprising. This work shows new surprise that 2 types of polymer nanoparticles exhibit self-sorting during its incorporation into a growing crystal. Mechanisms for the self-sorting have been discussed in association with the varied interfacial chemistry. I will suggest to accept this paper after minor revisions.

Reply: We sincerely thank Reviewer 2 for their positive evaluation of our work and particularly appreciate the acknowledgment of the unexpected nature of the self-sorting behavior observed during nanoparticle incorporation within growing crystals.

1. Self-sorting of polymers like molecular weight fractionation is quite well known (e.g., European Polymer Journal 107 (2018) 303–307). This work shows one type of it although the fractionation mechanism is quite different.

Reply: We thank the reviewer for this insightful comment. Indeed, self-sorting based on polymer molecular weight fractionation is well-established but the underlying mechanism differs from that reported herein. To acknowledge this important context, we have now briefly discussed the related work mentioned by the reviewer and cited the corresponding reference in our updated manuscript. The additional text (see pages 14-15) reads as follows: "*Other forms of self-sorting phenomena have also been reported. For example, in poly(ε-caprolactone) (PCL) spherulites, the degree of crystallinity exhibits a spatial distribution along the radial direction, with lower crystallinity observed at the spherulite center. Consequently, the central region of such spherulites melts at a lower temperature than that of the outer region.*[46]"

2. The self-sorting mechanism is associated with the varied interfacial chemistry. It might deserve to examine thermodynamic driving force (like varied solubility of the two nanoparticles) and the kinetic possibility (like varied sizes of the two nanoparticles). For the kinetics, it was described (e.g., Chernov, A. A. Modern Crystallography III 1984) that large particles get incorporated easier.

Reply: We thank the reviewer for these insightful comments. In our system, the two types of nanoparticles (M_{54}-B_{200} vesicles and S_{56}-B_{500} spheres) are both sterically stabilized diblock copolymer nanoparticles and are dispersed in aqueous solution. This is because both poly(methacrylic acid) (M_{54}) and poly(ammonium 2-sulfatoethyl methacrylate) (S_{56}) are anionic polyelectrolytes, highly hydrophilic and hence act as effective electrosteric stabilizers. Thus, from the perspective of solubility, no significant difference between the two nanoparticles is expected. However, our AFM studies reveal that M_{54} exhibits a stronger interaction with the calcite surface compared to S_{56}. From a thermodynamic standpoint, this suggests that M_{54}-based nanoparticles have a higher propensity to adsorb onto the growing crystal surface and subsequently undergo engulfment. This interpretation is consistent with prior studies led by J. J. De Yoreo and co-workers and L. A. Estroff et al. (see *Nat. Commun.* **2016**, *7*, 10187 and *J. Am. Chem. Soc.* **2018**, *140*, 7936-7945).

From a kinetic perspective, although particle size can influence the *extent* of occlusion, it does not affect the fundamental occlusion behavior. More specifically, increasing the particle size while maintaining the same surface

chemistry does *not* induce a transition from surface-confined occlusion to uniform occlusion. For example, increasing the mean diameter of poly(ammonium 2-sulfatoethyl methacrylate) stabilized nanoparticles from ~100 nm to ~200 nm does not alter their occlusion behavior; they remain preferentially occluded within the core region of the calcite crystals (see Supplementary Fig. 8f).

The above aspects are now discussed in three places in our updated manuscript (see page 10):

"*Nanoparticle occlusion within inorganic crystals is closely related to their ability to interact intimately with the growing crystal surface*[30,31], *with a strong surface interaction being essential for overcoming interfacial incompatibility and hence driving occlusion*[32]. *Both S_{56}-B_{500} spheres and M_{54}-B_{200} vesicles interact with calcite crystals through their respective polyelectrolytic stabilizer chains (S_{56} and M_{54}).*"

"*Notably, further experiments confirmed that the relatively large S_{30}-B_{300} vesicles (220 ± 73 nm diameter) were occluded within the central region of the calcite crystals (Supplementary Fig. 8), similar to the behavior observed for the relatively small S_{56}-B_{500} spheres (101 ± 27 nm diameter).*"

"*Given their similar degrees of polymerization, these AFM studies indicate that anionic M_{54} chains interact more strongly with calcite than S_{56} chains during crystallization.*"

3. The authors describe that 'M_{54}-B_{200} vesicles form large aggregates that cannot undergo occlusion'. It is not explained why aggregate cannot get incorporated. Actually, aggregates should get incorporated even easier according to refs (e.g., Chernov, A. A. Modern Crystallography III 1984).

Reply: We thank the reviewer for raising this important question. In our system, the key point is that M_{54}-B_{200} vesicles form macroscopic aggregates that under *sedimentation* at high Ca^{2+} concentrations (see Supplementary Fig. 6b). Under such conditions, these aggregates cannot effectively interact with the growing calcite crystals, which prevents their occlusion.

Accordingly, we have revised the text to clarify this mechanism, which now reads as follows on pages 12-13:

"The situation is more complex for M_{54}-B_{200} vesicles. At a relatively high Ca^{2+} concentration, M_{54}-B_{200} vesicles form large aggregates and precipitate, therefore occlusion cannot occur (state ②, Fig. 5). However, after a substantial reduction in the Ca^{2+} concentration, these flocculated vesicles undergo spontaneous redispersal to enable the belated occlusion of individual M_{54}-B_{200} vesicles (state ④, Fig. 5). This explains why M_{54}-B_{200} vesicles are predominantly located within the near-surface layer of the calcite crystals. The occlusion of M_{54}-B_{200} vesicles within calcite at such low Ca^{2+} concentrations is attributed to a stronger interaction between the M_{54} steric stabilizer chains and the calcite surface, as demonstrated by the force spectroscopy studies (Fig. 4). Indeed, when the initial Ca^{2+} concentration was reduced to 0.5 mM, M_{54}-B_{200} vesicles could be efficiently occluded, whereas S_{56}-B_{500} spheres were excluded from the calcite crystals (Supplementary Fig. 10)."

4. Language issues like 'syntheses'.

Reply: In response, the term "syntheses" has been replaced with "approaches" in the revised manuscript. The sentence on page 3 now reads: "*Notably, prior biomimetic approaches have typically focused on the interaction*

between single organic components and minerals…". In addition, the manuscript has been carefully proofread, and additional minor revisions (also yellow-highlighted) have been made to further improve clarity.

Reviewer 3:

The manuscript reports a new phenomenon involving particle occlusion within growing single crystals from a team with extensive expertise in this field. The results demonstrate that two distinct foreign particles can be incorporated into different regions of growing crystals, providing valuable insights into biomineralization and bio-inspired material synthesis. The findings are interesting and significant; however, I have several comments regarding the proposed mechanisms and interpretations.

Reply: We thank the reviewer for this positive and encouraging assessment of our work. We particularly appreciate the recognition of the novelty and significance of the observed self-sorting phenomenon, as well as its relevance to biomineralization and bio-inspired materials synthesis. We are grateful for the reviewer's constructive comments and have addressed the specific concerns regarding the proposed mechanisms and interpretations below.

1. For the TEM images shown in Fig. 1b, could the authors label one or two representative vesicles? Currently, it appears that many vesicles are cross-linked or aggregated together, making the structures difficult to distinguish.

Reply: We thank the reviewer for this helpful comment. The apparent aggregation of the vesicles in the TEM images simply arises from drying artifacts introduced during sample preparation. Indeed, our DLS studies confirm that these vesicles have a high degree of dispersion (i.e. no detectable aggregation) in aqueous solution.

As suggested, we have labeled several representative vesicles in the updated version of Fig. 1d using dashed circles to improve clarity. We have also explicitly clarified this point in the Fig. 1d caption (page 5), which now reads: "*TEM image of M_{54}-B_{200} vesicles. The dashed circles indicate individual vesicles. Their apparent aggregation is attributed to drying artifacts arising during TEM sample preparation.*"

2. More experimental details regarding the ammonia diffusion-controlled calcite growth should be provided in the SI.

Reply: We thank the reviewer for this constructive suggestion. The ammonia diffusion method is a well-established approach for the controlled growth of calcite crystals and has been extensively studied in the literature, particularly by Meldrum and co-workers (see *Ihli, et al. Adv. Funct. Mater.* ***2013****, 23, 1965-1973*). In response to the reviewer's request, we now include further experimental details in the "Methods" section. The updated text on page 22 reads as follows: "*Calcium carbonate crystals were prepared using the ammonia diffusion method*[29]*. A Petri dish containing 10 mL of a 5 mM $CaCl_2$ aqueous solution plus 0.05% w/w copolymer nanoparticles was placed in a desiccator. Glass slides (pre-cleaned using piranha solution) were positioned at the base of the Petri dish. Crystallization was induced by exposure to ammonium carbonate (2 g, placed at the bottom of the desiccator) for 24 h at 20 °C. Ammonium carbonate decomposes slowly to generate CO_2 and NH_3, which diffuse across the gas–liquid interface into the solution. Dissolved CO_2 reacts with water to form carbonic acid, which subsequently forms carbonate and bicarbonate ions, whose relative concentrations are governed by the solution pH. Concurrently,*

dissolved NH_3 increases the solution pH. In the presence of Ca^{2+} ions, these processes produce a solution that is supersaturated with respect to $CaCO_3$. After crystallization, the glass slides were removed, washed three times with deionized water, and rinsed three times with ethanol. Each occlusion experiment was repeated at least twice, with consistent results being obtained."

3. In the co-incorporation experiments, were the spheres already depleted from solution when the vesicles began to incorporate into the crystals?

Reply: This is a good question. Our experimental data indicate that the S_{56}-B_{500} spheres are *not* depleted from solution either prior to or during the incorporation of vesicles into the growing calcite crystals. This conclusion is supported by two independent lines of evidence. First, the in situ DLS measurements (Fig. 3d) clearly show that both types of nanoparticles remain detectable throughout the crystallization process, indicating their co-existence in solution. Second, the S_{56}-B_{500} aqueous dispersion exhibits a characteristic light pink color (see Supplementary Fig. 6a). Notably, this coloration persists even after crystallization is complete, providing further indirect evidence that these spherical nanoparticles remain in solution. In summary, our experimental observations indicate that self-sorting occlusion does not lead to the complete depletion of the spherical nanoparticles prior to vesicle incorporation.

Accordingly, we now include this point in the updated manuscript on page 8, where the text reads as follows: "*Furthermore, the in situ DLS data indicate that the characteristic population corresponding to the S_{56}-B_{500} spheres is always detected even during the latter stages of crystallization. This confirms that the S_{56}-B_{500} spherical nanoparticles are still present in solution during occlusion of the M_{54}-B_{200} vesicles within the growing crystals.*"

4. For the AFM force measurements, it is important to consider the loading rate (tip velocity). As the loading rate increases, the rupture force transitions from an equilibrium regime, which reflects bond/unbond events, to a kinetic regime where the measured force increases with loading rate. Therefore, the measurements should ideally be performed within the equilibrium regime to accurately determine binding energies. I assume the authors have considered this appropriately, but the relevant experimental details should be included in the SI. It is also suggested to present more representative force curves in SI.

Reply: We thank the reviewer for this insightful comment. We agree that the loading rate is a critical parameter in AFM force spectroscopy. In our experiments, the loading rate was carefully considered, and the measurements were performed within the equilibrium regime to ensure reliable determination of binding interactions. As suggested, we now include additional experimental details in the Supporting Information. More specifically, rupture force measurements were conducted at five different loading rates (20, 200, 1120, 2600, and 3910 nm s^{-1}), while maintaining a constant approach velocity of 200 nm s^{-1}. Our force spectroscopy data indicate an increase in mean rupture forces with increasing loading rate and the equilibrium force (f_{eq}) was obtained by fitting the force spectroscopy curves within the equilibrium regime during the entire stretching process. Accordingly, this information is now included in the updated Supporting Information and a new Supplementary Fig. 7 has been added (see below).

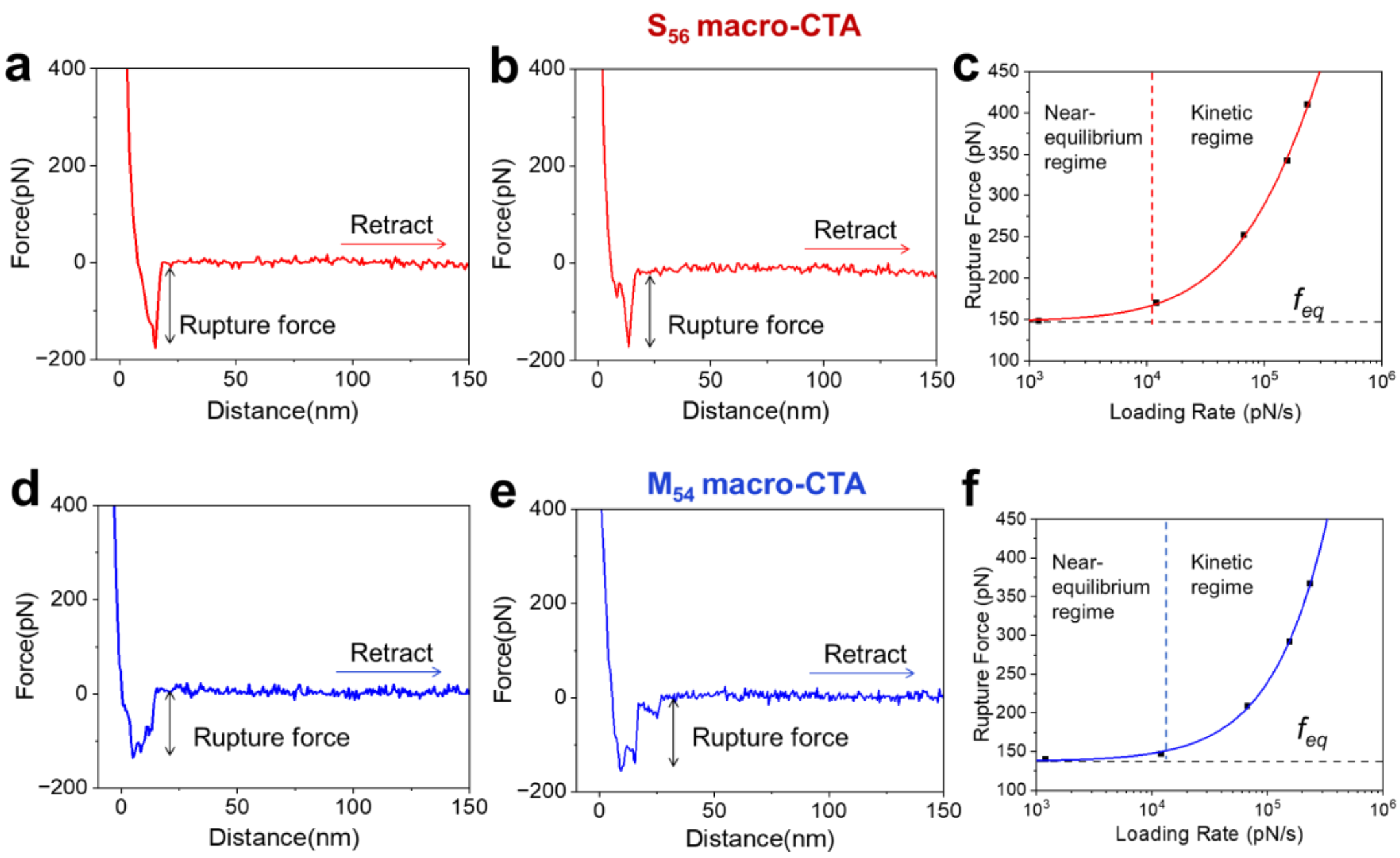

Supplementary Fig. 7. AFM-based force spectroscopy measurements of the interaction between the steric stabilizer chains (either S_{56} or M_{54}) and the calcite surface. The modified AFM tip functionalized with (a-b) S_{56} or (d-e) M_{54} chains was forced to repeatedly advance towards and retreat from the calcite surface to obtain a series of representative force-distance curves. (c, f) Force spectroscopy data indicate an increase in rupture force with increasing loading rate; the dashed lines indicate the equilibrium force (f_{eq}) required to detach the S_{56} or M_{54} chains from the calcite surface.

5. On page 11, the authors introduce three additional particles (e.g., $S_{30}B_{300}$ and $M_{36}B_{200}$). How do the hydrodynamic diameters of these particles compare with those of the original systems? In addition, a transition sentence introducing this new set of experiments would improve the readability of the paragraph.

Reply: We thank the reviewer for this question and helpful suggestion. The purpose of introducing these additional particles was to further demonstrate that neither the size of the guest nanoparticles nor the degree of polymerization (DP) of the steric stabilizer block significantly influences their occlusion behavior. To clarify the size differences more explicitly, we have revised the corresponding figures. In the updated version, we have included the hydrodynamic diameter of the S_{30}-B_{300} nanoparticles and performed pH- and [Ca^{2+}]-dependent titration experiments. The latter data indicate that these nanoparticles exhibit excellent colloidal stability under the same conditions as the S_{56}-B_{500} nanoparticles (see revised Supplementary Fig. 8b-c). More specifically, the S_{30}-B_{300} nanoparticles have a hydrodynamic diameter of 220 ± 73 nm, which is larger than that of the S_{56}-B_{500} nanoparticles (101 ± 27 nm). Despite this size difference, S_{30}-B_{300} displays the same occlusion behavior as S_{56}-B_{500}, thus confirming that nanoparticle size is not a critical parameter in determining whether occlusion occurs or not.

Similarly, the hydrodynamic diameters of M_{36}-B_{200} and M_{85}-B_{300} vesicles are 220 ± 38 nm and 342 ± 99 nm, respectively, compared to a hydrodynamic diameter of 306 ± 92 nm for M_{54}-B_{200}. Although these vesicles differ in both size and the mean DP of the poly(methacrylic acid) stabilizer chains, they invariably undergo surface-confined occlusion (see revised Supplementary Fig. 9). This suggests that neither particle size nor stabilizer chain length plays a decisive role in determining the occlusion mode.

Accordingly, we have improved the logical flow of the manuscript text by introducing this set of experiments with an appropriate explanatory sentence. The revised text now reads (pages 10): "*Notably, further experiments confirmed that the relatively large S_{30}-B_{300} vesicles (220 ± 73 nm diameter) were occluded within the central region of the calcite crystals (Supplementary Fig. 8), similar to the behavior observed for the relatively small S_{56}-B_{500} spheres (101 ± 27 nm diameter). This observation indicates that the occlusion behavior of these sulfate-based nanoparticles within calcite crystals is governed neither by the degree of polymerization of poly(ammonium 2-sulfatoethyl methacrylate) nor by particle size. This observation indicates that the occlusion behavior of these sulfate-functionalized nanoparticles within calcite crystals is governed neither by the mean DP of their poly(ammonium 2-sulfatoethyl methacrylate) steric stabilizer chains nor by their particle size. Furthermore, the mean DP of the poly(methacrylic acid) chains also does not influence the occlusion behavior. Both M_{36}-B_{200} (220 ± 38 nm) and M_{85}-B_{300} (342 ± 99 nm) vesicles, which comprise shorter and longer steric stabilizer chains respectively, are incorporated as a surface-confined layer within growing calcite crystals (Supplementary Fig. 9), which is consistent with the behavior observed for M_{54}-B_{200} vesicles.*"

6. Assuming that the carbonate concentration remains relatively constant due to equilibration with atmospheric CO_2, the decrease in calcium concentration during growth would reduce the solution supersaturation and consequently decrease the calcite growth rate (e.g., step velocity). This introduces an important kinetic factor that should be considered in the discussion.

Reply: We thank the reviewer for this insightful comment highlighting the important role of supersaturation and growth kinetics during calcite crystallization. We agree that the reduction in Ca^{2+} concentration during crystal growth lowers the solution supersaturation and consequently leads to a slower rate of calcite growth. In our study, we performed control experiments at $[Ca^{2+}]$ = 0.5 mM in the presence of either S_{56}-B_{500} spheres or M_{54}-B_{200} vesicles (Supplementary Fig. 10). Under such conditions, no occlusion of S_{56}-B_{500} spheres occurred, whereas M_{54}-B_{200} vesicles were still incorporated into the calcite crystals. These observations are consistent with our *in situ* observations during self-sorting occlusion (Fig. 3).

Our results demonstrate that the calcite growth rate alone does not determine the outcome. More specifically, for experiments conducted at lower initial Ca^{2+} concentrations—where crystal growth is inevitably slower—M_{54}-B_{200} vesicles are occluded while S_{56}-B_{500} spheres are excluded. This striking difference is attributed to their differing interfacial interactions with calcite, as indicated by AFM force spectroscopy experiments. These measurements confirm that the M_{54} chains interact more strongly with calcite than the S_{56} chains. Consequently, the S_{56}-B_{500} spheres are less readily incorporated at lower $[Ca^{2+}]$ concentration. In contrast, the M_{54}-B_{200} vesicles are less sensitive to calcite growth kinetics but susceptible to colloidal instability at a relatively high ionic strength ($[Ca^{2+}]$ = 5 mM). These findings highlight the subtle interplay between kinetic factors and interfacial interactions in governing the self-sorting occlusion behavior. Accordingly, these points have now been incorporated into the updated manuscript at the relevant locations (see new yellow-highlighted text on pages 11-13).

7. How do the relative bulk (and interfacial) diffusivities of the two particles compare? Since the vesicles are much larger and also appear prone to aggregation/cross-linking, one would expect them to possess substantially lower diffusivity. In this context, the thermodynamic parameters measured by AFM may not fully explain the observed behavior. Considering the simultaneous decrease in crystal growth kinetics, it may be possible to interpret the self-sorting phenomenon from a kinetic-control perspective: the smaller spheres, with faster diffusion and/or lower

kinetic barriers, preferentially incorporate during initial periods of higher growth rate, whereas the larger vesicles incorporate later under slower growth conditions.

Reply: We thank the reviewer for this insightful and constructive comment. We particularly appreciate the reviewer's effort to rationalize the self-sorting mechanism from both thermodynamic and kinetic perspectives. First, we would like to clarify that the AFM measurements in this study were designed to quantify the interfacial interactions between the steric stabilizer chains (S_{56} or M_{54}) and the calcite (104) surface. These experiments demonstrate that the M_{54} chains interact more strongly with calcite compared to the S_{56} chains. We agree with the reviewer that, in principle, larger nanoparticles (e.g. the M_{54}-B_{200} vesicles) will diffuse more slowly than smaller nanoparticles (e.g. the S_{56}-B_{500} spheres). However, our control experiments indicate that particle size—and by implication diffusivity—is not the decisive factor governing spatially-confined occlusion. For example, relatively large S_{30}-B_{300} nanoparticles (220 ± 73 nm diameter) are occluded within the central region of calcite crystals, in the same manner as the smaller S_{56}-B_{500} nanoparticles (Supplementary Fig. 8f). This observation indicates that particle size alone does not dictate the occlusion mode. Instead, the observed self-sorting behavior arises from the subtle interplay between colloidal stability and interfacial interactions, both of which evolve during the crystallization process as the Ca^{2+} concentration is progressively reduced.

The absence of the M_{54}-B_{200} vesicles from the calcite crystal cores is a direct consequence of their colloidal instability at the relatively high initial Ca^{2+} concentration of 5 mM. However, as crystallization proceeds and the Ca^{2+} solution concentration is lowered, the flocculated vesicles spontaneously redisperse, hence enabling their subsequent incorporation into the outer regions of the calcite crystals. In contrast, the S_{56}-B_{500} spheres remain well-dispersed throughout the crystallization process. Consequently, their occlusion can occur even during the early stages of crystal growth. However, AFM measurements indicate that the S_{56} chains interact more weakly with the calcite surface compared to the M_{54} chains. As the rate of calcite formation is reduced at lower Ca^{2+} concentration, this weaker interaction becomes insufficient to sustain occlusion, thus leading to exclusion of the S_{56}-B_{500} nanoparticles in the latter stages. In summary, the observed occlusion behavior is primarily dictated by the dynamic balance between colloidal stability and polymer–crystal interfacial interactions, which together determine the temporal window during which each type of nanoparticle can be incorporated.

8. I am somewhat confused by the new experiments introduced immediately before the Discussion section involving microparticles. The results are interesting and appear to show some degrees of similar self-sorting behavior. However, because the spheres are already attached to the microparticles, would they still undergo the same aggregation–dispersion processes discussed previously? If not, the earlier explanation based on the colloidal stability of vesicles appears somewhat contradictory and may require clarification.

Reply: We thank the reviewer for highlighting a potential source of confusion. These additional experiments were performed to examine our hypothesis that self-sorting occlusion is governed by surface chemistry, which in turn dictates both colloidal stability and interfacial interactions. To this end, we designed "core–satellite" composite microparticles by adsorbing either sulfonate-based or carboxylate-based diblock copolymer nanoparticles onto larger substrates (MSNs-NH_2 or UiO-66-NH_2). Although these nanoparticles are immobilized at the surface of the microparticles, the key point is that the interfacial chemistry presented to the crystallizing calcite is still dictated by

the outer layer of adsorbed nanoparticles. Importantly, we find that these composite microparticles exhibit aggregation–dispersion behavior that closely resembles that of the corresponding free nanoparticles over a wide range of Ca^{2+} concentration (see new Supplementary Fig. 13c below). Consequently, their occlusion behavior is governed by the nature of the surface-bound nanoparticles.

Experimentally, this is confirmed by the following observations. Firstly, uncoated MSNs-NH_2 and UiO-66-NH_2 microparticles do not undergo occlusion (Supplementary Fig. 14). In contrast, such microparticles are incorporated into calcite crystal cores when decorated with sulfonate-based nanoparticles, whereas the microparticles decorated with carboxylate-based nanoparticles are solely incorporated into the near-surface regions of calcite crystals. Secondly, when both types of nanoparticle-coated microparticles are present in the reaction solution, there is clear evidence for self-sorting occlusion behavior (Supplementary Fig. 15).

We acknowledge that our original manuscript did not clearly articulate this rationale, which no doubt led to Reviewer 3's confusion. Therefore, we have revised the relevant section of text (see page 13), which now reads as follows:

"*To examine whether self-sorting occlusion is a generic phenomenon, we performed further experiments. Given that the occlusion behavior of guest nanoparticles is closely governed by their surface chemistry, we posited that, if relatively large guest microparticles were coated with nanoparticles, then the occlusion behavior of the former system should reflect that of the latter. To examine this hypothesis, two new types of diblock copolymer nanoparticles were prepared via RAFT-mediated PISA…*".

The new Supplementary Fig. 13 is shown overleaf.

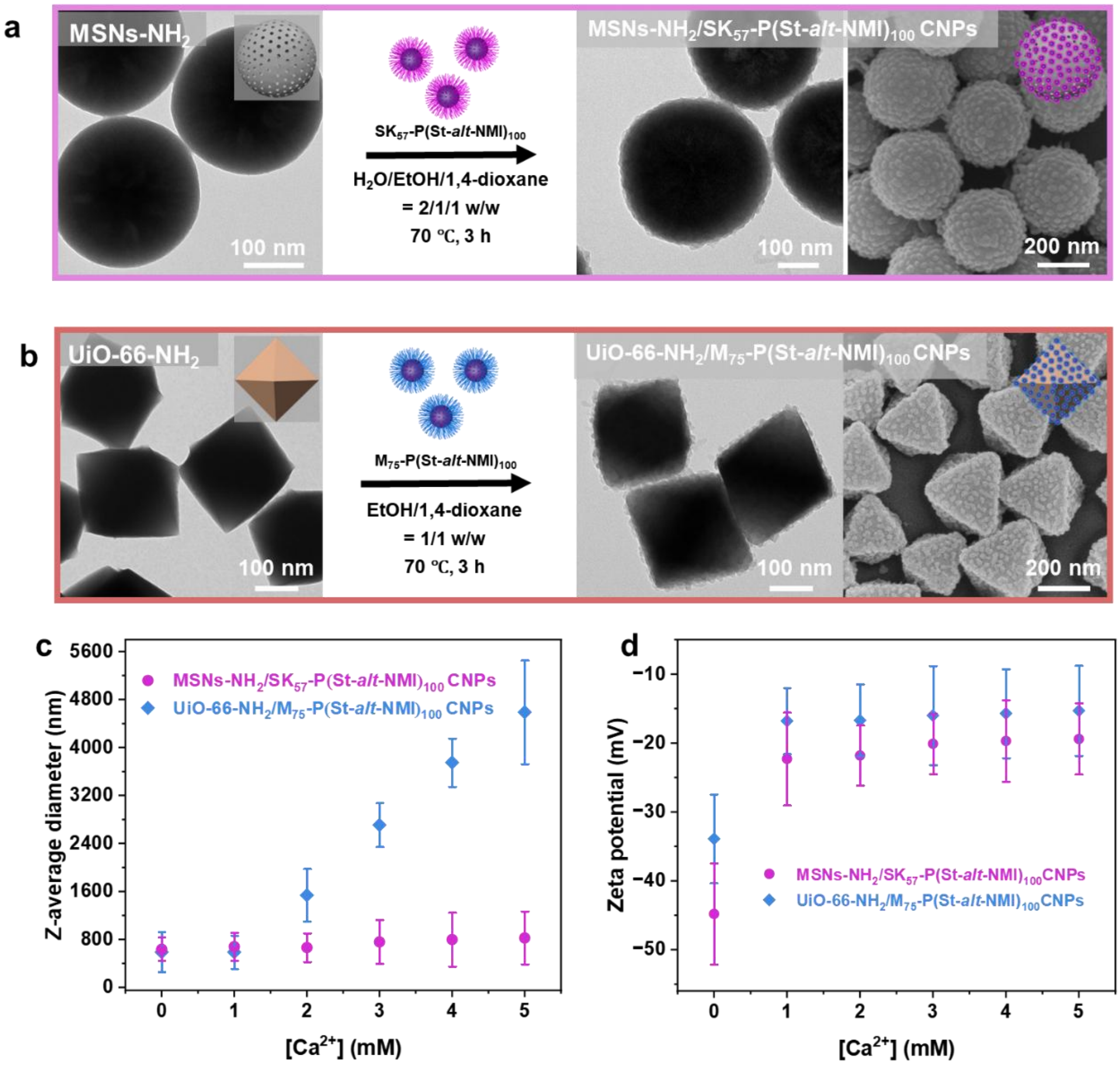

Supplementary Fig. 13. Preparation and [Ca^{2+}]-dependent colloidal stability of composite nanoparticles (CNPs) by surface decoration of spherical MSNs-NH_2 and octahedral UiO-66-NH_2 with SK_{57}-P(St-*alt*-NMI)$_{100}$ and M_{75}-P(St-*alt*-NMI)$_{100}$, respectively. a, Synthesis and characterization of MSNs-NH_2/SK_{57}-P(St-*alt*-NMI)$_{100}$ CNPs; **b,** Synthesis and characterization of Uio-66-NH_2/M_{55}-P(St-*alt*-NMI)$_{100}$ CNPs; **c,** Hydrodynamic diameters as a function of Ca^{2+} concentration as determined by DLS; **d**, Zeta potential data as a function of Ca^{2+} concentration.

9. The authors mainly attribute the self-sorting behavior to changes in colloidal stability arising from variations in calcium concentration. One possible way to directly test this hypothesis would be to use a flow-cell setup to maintain a constant calcium concentration during crystal growth. This is only a suggestion and not intended as a required experiment, but it may help strengthen the mechanistic interpretation.

Reply: We thank the reviewer for this insightful and constructive suggestion. We agree that a flow-cell setup would provide a powerful approach to directly probe the occlusion behavior of the two types of guest nanoparticles under a known constant calcium ion concentration, thereby enabling a more rigorous assessment of the proposed mechanism. However, we emphasize that the combined *in situ* DLS measurements and SEM observations presented in Fig. 3 already provide indirect but compelling evidence for the role of calcium ion concentration. By correlating the temporal evolution of particle size distributions (from DLS) with the spatial distribution of occluded particles

(from SEM), we effectively capture the behavior of the two nanoparticle populations at different stages of crystallization, which corresponds to the progressive reduction in the Ca^{2+} concentration. Hence we demonstrate that the occlusion behavior of M_{54}-B_{200} vesicles is strongly governed by their colloidal stability, which is highly sensitive to the instantaneous calcium ion concentration. In contrast, the S_{56}-B_{500} spheres remain colloidally stable across the entire range of calcium concentrations investigated herein, and their occlusion is instead primarily dictated by the strength of their interfacial interaction with the growing calcite crystals. We are currently exploring related systems and plan to implement such a flow-cell approach to further validate and extend these findings.

In addition to the above changes, we have also proofread the manuscript one more time and made a few minor adjustments to the text to improve its clarity (with all such changes also indicated via yellow-highlighted text). Moreover, a few additional highly relevant just-published references have been cited in the revised manuscript.

Finally, we sincerely thank these three reviewers again for their insightful comments and valuable suggestions.

Reviewer 3:

The authors have well addressed my comments and have thoroughly revised the manuscript. The new data and text now clearly support the conclusion that the proposed self-sorting phenomenon is governed by interfacial chemistry rather than particle size or topology. I have one remaining minor comment; otherwise, I believe the manuscript is suitable for publication.

The force spectroscopy was performed at low supersaturation, corresponding to the low-Ca-concentration regime rather than the initial high-Ca regime. The zeta potential data in Figure S6 show that the potential decreases with increasing Ca concentration, becoming less negative for both types of particles. Could the authors clarify why the interaction between S_{56} chains and the calcite surface increases with increasing Ca concentration? Is this enhancement primarily due to changes in the zeta potential of the calcite surface (and the particle), or does it reflect differences in chemical bonding strength?

Reply: We thank the reviewer for raising these insightful points.

We would like to clarify that the AFM force spectroscopy measurements were not performed over a range of Ca^{2+} concentrations. Instead, all force measurements were conducted in a calcite-supersaturated solution (σ = 0.274), corresponding to near-equilibrium crystal-growth conditions. Consequently, the force spectroscopy experiments do not establish a Ca^{2+}-dependent trend in the interaction between the polymer chains and the calcite surface. By contrast, the zeta potential measurements shown in Fig. S6d were obtained independently as a function of Ca^{2+} concentration to probe changes in the interfacial electrostatic environment. Compared with M_{56}-B_{200} vesicles, S_{56}-B_{500} spheres exhibit consistently less negative zeta potentials over the entire Ca^{2+} concentration range and a more pronounced shift toward charge neutralization with increasing Ca^{2+} concentration. This behaviour indicates that Ca^{2+} more effectively modifies the electrostatic environment surrounding the sulfate-functionalized corona, most likely through enhanced counterion accumulation and electrical double-layer compression. Importantly, these observations reflect differences in electrostatic screening and do not provide direct evidence for stronger specific Ca^{2+}–polymer coordination or intrinsically stronger interactions between the S_{56} chains and the calcite surface.

Instead, our proposed mechanism is that Ca^{2+} primarily acts as an ionic bridge between the negatively charged sulfate groups of the S_{56} chains and the negatively charged calcite surface. Thus, although our AFM measurements do not directly probe the dependence of interaction force on Ca^{2+} concentration, a higher Ca^{2+} concentration is expected to promote Ca^{2+}-mediated bridging and thereby enhance the effective interaction between the S_{56} chains and the calcite surface. As crystallization proceeds, however, the Ca^{2+} concentration decreases substantially, reducing the availability of Ca^{2+} ions to mediate this bridging interaction. Under these conditions, the relatively weak intrinsic interaction between the S_{56} chains and the calcite surface, as demonstrated by the AFM force spectroscopy measurements (Fig. 4e), becomes dominant. Consequently, S_{56}-B_{500} spheres exhibit only limited affinity for the growing calcite surface and are no longer efficiently incorporated during the later stages of crystal growth. As a result, S_{56}-B_{500} spheres are predominantly retained within the crystal cores, having been occluded during the initial stage of crystallization.

To avoid potential misunderstanding, we have added the following clarification after Supplementary Fig. S6:

"It should be emphasized that the AFM force measurements and zeta potential measurements were conducted under different experimental conditions and therefore should not be directly correlated as a function of Ca^{2+} *concentration."*

In addition, we have revised the main text to further clarify the role of Ca^{2+}-mediated ionic bridging during different stages of crystal growth. The revised text reads:

"*However, during the latter stages of calcite growth, the* Ca^{2+} *concentration decreases substantially (e.g., below 1 mM), weakening the* Ca^{2+}*-mediated ionic bridging (state ③, Fig. 5). Moreover, the intrinsic interaction between the* S_{56} *chains and the calcite surface is relatively weak compared with that of the* M_{54} *chains, as demonstrated by the AFM force measurements (Fig. 4e). Consequently, once the ionic bridging is diminished,* S_{56}*-*B_{500} *spheres exhibit only limited affinity for the growing calcite surface and are no longer efficiently incorporated during subsequent crystal growth. As a result,* S_{56}*-*B_{500} *spheres are predominantly retained within the crystal cores, having been occluded during the early stages of crystallization.*"

Many thanks again for your insightful comments, which really strengthened this manuscript.
