# Supplementary material for: Interfacial chemistry governs nanoparticle self-sorting during biomimetic crystallization
Source: Nat Commun. 2026 Aug 4;17:9382. doi: 10.1038/s41467-026-75685-3 (PMC13534651; doi:10.1038/s41467-026-75685-3)
Supplement: Supplementary file 3 [file 41467_2026_75685_MOESM1_ESM.docx]

**Supplementary Information for**

**Interfacial chemistry governs nanoparticle self-sorting during biomimetic crystallization**

Wenting Chen (陈雯婷),^1,2^ Zhuodi Fan (范卓迪),^1,2^ Pei Liu (刘配),^1,2^ Xiaohong Hu (胡小红),*^,1,2,3^ Yihao Yang (杨怡豪),^1,2^ Qin Li (李琴),^1,2^ Meijiang Wang (王美江),^1,2^ Jingjing He (何晶晶),^1,2^ Wenjun Zhang (张文君)*^, 4^, Steven P. Armes,*^,5^ and Yin Ning (宁印)*^,1,2,3^

^1^ State Key Laboratory of Bioactive Molecules and Druggability Assessment, College of Chemistry and Materials Science, Jinan University, Guangzhou 510632, China.

^2^ Guangdong Provincial Key Laboratory of Supramolecular Coordination Chemistry, College of Chemistry and Materials Science, Jinan University, Guangzhou 510632, China.

^3^ Guangdong Basic Research Center of Excellence for Natural Bioactive Molecules and Discovery of Innovative Drugs, College of Chemistry and Materials Science, Jinan University, Guangzhou 510632, China.

^4^ College of Resources and Environment, Huazhong Agricultural University, Wuhan 430070, China.

^5^ School of Mathematical and Physical Sciences, University of Sheffield, Brook Hill, Sheffield, South Yorkshire S3 7HF, UK.

**E-mail:** xiaohonghu@jnu.edu.cn (X.H.); wenjunzhang@mail.hzau.edu.cn (W.Z.); s.p.armes@sheffield.ac.uk (S.P.A.); yinning@jnu.edu.cn (Y.N.)

1. **Experimental Section**

***1.1. Materials***

Methacrylic acid (MAA, ≥ 99%), 3-sulfopropyl methacrylate potassium (SK, 99%) and styrene (St, ≥ 99%) were purchased from Macklin (Shanghai, China). Benzyl methacrylate (BzMA, 98%) was obtained from Konoscience (Beijing, China) and ammonium 2-sulfatoethyl methacrylate (SEM, supplied as a 25% w/v aqueous solution) was obtained from GEO Specialty Chemicals (Hythe, UK). *N*-Phenylmaleimide (NMI, 98%) was purchased from Alfa and recrystallized from cyclohexane at 90 °C before use. Cyano-4-(phenylcarbonothioylthio)pentanoic acid (CPCP), 3-aminopropyltriethoxysilane (APTES, 98%) and zirconium tetrachloride (ZrCl_4_, 98%) were obtained from Meryer (Shanghai, China). 2-(Methacryloyloxy)ethyl thiocarbamoyl rhodamine B (RhBMA) and fluorescein O-methacrylate (FMA, 97%) were purchased from Sigma-Aldrich. 4,4′-Azobis(4-cyanovaleric acid) (ACVA, 98%) and ammonium carbonate ((NH_4_)_2_CO_3_, 30% NH_3_ basis) were purchased from Aladdin (Shanghai, China). Calcium chloride hexahydrate (CaCl_2_·6H_2_O, 98%) was supplied by OKA (Beijing, China). Cetyltrimethylammonium bromide (CTAB, ≥96%), ammonium hydroxide solution (25–28%), tetraethyl orthosilicate (TEOS, 98%), 2-amino-1,4-benzenedicarboxylic acid (BDC-NH_2_, 98%), 1,4-dioxane (99%), and *N*,*N*-dimethylformamide (DMF, 99%) were purchased from Macklin (Shanghai, China). Acetic acid (99%), methanol (99%) and ethanol (99%) were supplied by Guangzhou Chemical Reagent Factory (Guangzhou, China). Deionized water (18.25 MΩ·cm) was obtained from a Milli-Q water purification system.

***1.2. Synthesis of poly(3-sulfopropyl methacrylate potassium)_57_ macromolecular chain transfer agent (SK_57_ macro-CTA)***

A round-bottomed flask was charged with CPCP (340.3 mg, 1.2 mmol), ACVA initiator (68.3 mg, 0.24 mmol; [CPCP]/[ACVA] molar ratio = 5.0), and SK (15.0 g, 60.9 mmol), followed by the addition of 3:1 w/w water/methanol (23 g). The solution pH was adjusted to pH 6 using 0.10 M HCl. The flask was sealed with a rubber septum, degassed under nitrogen for 30 min in an ice–water bath, and then transferred to a preheated oil bath at 70 °C for 3 h. The polymerization was quenched by cooling the mixture to room temperature (25 °C) and exposing the reaction mixture to air. The crude product was purified by four successive precipitations into a ten-fold excess of ethanol, followed by redissolution in water. The final macro-CTA was obtained as a pink powder after lyophilization. The mean DP of 57 was determined by end-group analysis using ^1^H NMR spectroscopy by comparing the integrated intensity of the aromatic protons at 7.2-8.0 ppm to the methacrylic backbone protons at 0.5-2.5 ppm.

***1.3. Synthesis of poly(3-sulfopropyl methacrylate potassium)_57_-block-poly(styrene-alt-N-phenylmaleimide)_100_ [SK_57_-P(St-alt-NMI)_100_] spheres***

SK_57_ macro-CTA (286.4 mg, 20 μmol), ACVA initiator (1.86 mg, 6.7 μmol; [SK_57_ macro-CTA]/[ACVA] molar ratio = 3.0), St (104.2 mg, 1.0 mmol), and NMI (173.2 mg, 1.0 mmol) were dissolved in 5.1 g of a ternary solvent mixture comprising 50:25:25 water/ethanol/1,4-dioxane by mass to afford 10% w/w solids content. The solution was transferred to a sealed flask, degassed using a stream of nitrogen gas for 20 min at 25 °C, and subsequently heated in an oil bath at 70 °C for 24 h to achieve high comonomer conversion (>99%). The copolymerization was quenched by cooling the flask in an ice–water bath and exposing the reaction solution to air.

***1.4. Synthesis of poly(methacrylic acid)_75_-block-poly(styrene-alt-N-phenylmaleimide)_100_ [M_75_-P(St-alt-NMI)_100_] spheres***

M_75_ macro-CTA (134.7 mg, 20 μmol), ACVA initiator (1.86 mg, 6.6 μmol; [M_75_ macro-CTA]/[ACVA] molar ratio = 3.0), St (104.2 mg, 1.0 mmol), and NMI (173.2 mg, 1.0 mmol) were dissolved in 50:50 w/w ethanol/1,4-dioxane (3.7 g) to afford 10% w/w solids content. The solution was transferred to a flask, which was sealed and degassed using a stream of nitrogen gas for 20 min at 0 °C. The flask was then immersed in an oil bath at 70 °C for 24 h to achieve high comonomer conversion (>99%). The copolymerization was quenched by cooling the flask in an ice–water bath and exposing the reaction solution to air.

***1.5. Synthesis of amine-modified meso-porous silica nanoparticles (MSNs-NH_2_)***

CTAB (1.0 g, 2.7 mmol), deionized water (125 mL), ethanol (75 mL), and ammonium hydroxide solution (1.25 mL) were added to a round-bottom flask under vigorous stirring. TEOS (1.3 mL, 5.8 mmol) was then added dropwise to the mixture, after which the flask was sealed and stirred at room temperature until a milky white suspension was obtained. After 24 h, the product was treated with 52 mL of a 50/2 v/v ethanol/hydrochloric acid solution to remove excess CTAB, followed by three washing cycles with deionized water and ethanol via centrifugation. The resulting MSNs (0.63 g) were dispersed in ethanol (20 g) with ammonium hydroxide solution (0.5 mL) in a round-bottom flask and then APTES (4.5 mL, 19.2 mmol) was added. The mixture was stirred at 80 °C for 12 h, after which the amine-modified MSNs (MSNs–NH_2_) were purified by three centrifugation cycles (3,500 rpm, 1259 ×g, 8 min per cycle) using ethanol.

***1.6. Synthesis of zirconium-based metal-organic frameworks (UiO-66-NH_2_)***

ZrCl_4_ (102 mg, 0.4 mmol) and BDC-NH_2_ (145.0 mg, 0.8 mmol) were each dissolved separately in DMF (50 mL). The two solutions were then combined in a flask, followed by the addition of glacial acetic acid (12 mL). The flask was sealed and the reaction was allowed to proceed without stirring at 120 °C for 3 h. The resulting product was collected by centrifugation (9000 rpm, 8326 ×g, 5 min), washed three times with DMF, and finally dispersed in DMF to afford a 10% w/w dispersion.

***1.7. Synthesis of MSNs-NH_2_/SK_57_-P(St-alt-NMI)_100_ hybrid particles***

MSNs-NH_2_ (90 mg) was dispersed in a ternary mixture of 50:25:25 H_2_O/ethanol/1,4-dioxane by mass (2.0 g). This dispersion was then added dropwise to a flask containing SK_57_-P(St-*alt*-NMI)_100_ (45 mg) dispersed in the same solvent (8.0 g). The mixture was stirred at 70 °C for 3 h, after which the colloidal nanocomposite particles (CNPs) were isolated by three centrifugation–redispersion cycles (6000 rpm, 3700 ×g, 5 min per cycle) against water. Same protocol was employed to make UiO-66-NH_2_/M_75_-P(St-alt-NMI)_100_ hybrid particles.

2. ***Characterization***
   2.1. ***^1^H NMR spectroscopy***

All ^1^H NMR spectra were recorded on a Bruker Avance 400 MHz spectrometer at 25 °C, using D₂O, CD₃OD, or DMSO-d₆ as solvents, with 64 scans collected for each spectrum.

2.2. ***Dynamic light scattering (DLS)***

DLS measurements were performed using a Malvern Zetasizer NanoZS instrument, which detects backscattered light at an angle of 173°. Aqueous electrophoresis measurements were conducted with disposable folded capillary cells (DTS1070, Malvern) using the same instrument. Copolymer nanoparticle dispersions were diluted to 0.10% w/v with deionized water. For pH titrations, the dispersion was initially adjusted to pH 11 using 0.5 M NaOH and then titrated by incremental addition of 0.01 M or 0.1 M HCl. For Ca^2+^ titration experiments, the nanoparticle concentration was fixed at 0.10% w/w, while the Ca^2+^ concentration was varied (0, 0.25, 0.50, 0.75, 1.00, 2.00, 3.00, 4.00, 5.00, or 6.00 mM) at approximately pH 9. Samples were equilibrated for 60 s prior to measurement. Each measurement consisted of 10-15 runs, and three measurements were performed for each sample. Data are reported as the mean ± standard deviation from three independently prepared samples.

2.3. ***Transmission electron microscopy (TEM)***

TEM samples were prepared by depositing 10 μL droplets of a 0.10% w/v aqueous dispersion of copolymer nanoparticles onto carbon-coated palladium–copper grids (Sigma, China). Prior to sample deposition, the copper grids were treated by plasma glow discharge for approximately 40 s to render the surface hydrophilic. A droplet (10 μL) of the aqueous nanoparticle dispersion was then placed onto the grid, and after allowing it to rest for 30 s, the excess sample was gently blotted away. For S_56_-B_500_ nanoparticle spheres, additional negative staining was performed on the copper mesh background using 8 μL of phosphotungstic acid solution (2%, pH 7.0, OKA), applied for 30 s and subsequently removed by blotting, to enhance the electron contrast. TEM imaging was conducted using a JEOL JEM-1400 Flash microscope operating at 120 kV.

2.4. ***Confocal laser scanning microscopy (CLSM)***

CLSM imaging of the composite crystals was performed on a Zeiss LSM 588 microscopy. Fluorescence was excited at 488 nm for RhBMA and at 566 nm for FMA, and emission was collected at 580 nm and 506 nm, respectively. Images were acquired using a 60× oil-immersion objective and processed with ZEN Lite 2020 software. Nanoparticle release from the composite crystals was examined under acidic conditions. The glass slide bearing the composite crystals was mounted in a microscopy observation chamber, and an initial image (0 min) was recorded after focusing. Subsequently, 2.5 mL of an acidic buffer (potassium hydrogen phthalate, pH 4.00) was gently added to the chamber. CLSM images were collected every 5 min until the crystals were fully etched. Before each acquisition, the focal plane was slightly readjusted, as the carbon dioxide generated during the acid-etching process caused slight changes in the position and height of the specimen.

2.5. ***Scanning electron microscopy (SEM)***

Randomly fractured composite crystals were prepared by placing a clean glass slide over the crystal-bearing slide and gently pressing and twisting to break the crystals. The resulting fragments were sputter-coated with gold (10 mA, 40 s) and imaged on a Zeiss Ultra 55 SEM operated at 3.0 kV with a 50 μA beam current. To obtain flat cross-sections and visualize the internal distribution of S_56_-B_500_ spheres and M_54_-B_200_ vesicles, the crystals were subjected to argon ion beam etching (PECS II Model 685; 6.0 kV; 5 h) and subsequently examined using a Thermo Scientific Apreo 2S SEM operated at 2.0 kV with a 13 μA beam current without metal coating.

2.6. ***Force spectroscopy experiments using an atomic force microscope (AFM)***

Gold (Au)-coated AFM tips were functionalized by incubating with 50 μg/L PMAA_54_ or PSEM_56_ solutions for 12 h, during which the terminal thioester groups covalently anchored the anionic polymer chains to the Au surface. The modified tips were subsequently rinsed twice with ultrapure water to remove any unbound polymer. Force spectroscopy experiments were conducted in force-volume mode using a Nanoscope V-Multimode 8 AFM (Bruker, Santa Barbara, CA) to probe the interactions between the polymer-functionalized tips and calcite (10$\bar{1}$4) surfaces within a supersaturated solution with respect to calcite (*σ* = 0.274), which represents near-equilibrium conditions for calcite growth^1,2^. The sensitivity and spring constants of the polymer-modified tips were calibrated prior to measurements. Force–distance curves were recorded at an approach velocity of 200 nm/s and different loading rates including 20, 200, 1120, 2600 and 3910 nm/s. Data were analyzed using NanoScope Analysis software (Bruker). Rupture forces were determined, and the contour lengths of the stretched polymers were obtained by fitting the force–extension curves using a worm-like chain (WLC) model:

$$\begin{aligned} \frac{fl_{\mathrm{app}}}{k_{B}T}=\frac{x}{L}+\frac{1}{{4\left( 1-\frac{x}{L} \right)}^{2}}-\frac{1}{4} \end{aligned}$$

where $f$ represents the rupture force, $l_{\mathrm{app}}$ is the apparent persistence length, which describes the slope of the force-distance curve. $L$ stands for the contour length of the polymer and $x$ is the rupture distance between the modified tip and the calcite (10$\bar{1}$4) surfaces. The WLC model provides a quantitative framework to analyze the force-extension behavior of polymers, allowing for the extraction of key parameters such as the persistence length and contour length.

2.7. ***In situ kinetic analysis of the occlusion of spheres and vesicles during calcite crystallization***

A Cordouan Vasco Kin particle analyzer (Cordouan Technologies Ltd., Pessac, France), equipped with an *in situ* head detector, was employed to monitor changes in the particle size distribution, which was calculated using Nankin software. This system enabled real-time tracking of the relative consumption of spheres and vesicles during the calcite crystallization process.

2.8. ***Inductively coupled plasma optical emission spectroscopy (ICP-OES)***

The concentration of free Ca^2+^ at different time points was determined using a Thermo X Series 2 ICP-OES instrument under an HCl matrix of 0.2 mol L^-1^. Prior to analysis, the samples were filtered through a 0.22 μm aqueous membrane to remove organic particulates and large aggregates. The filtrates were then fully acidified with concentrated hydrochloric acid and subsequently diluted 1:1 with deionized water. To ensure complete removal of residual organic matter, the solutions were passed through an activated RP pretreatment column. Each measurement was performed at least in duplicate.

2.9. ***Other measurements***

Optical microscopy images were obtained using an Olympus microscope equipped with an AxioCam 1Cm1 monochrome camera. Raman spectra were recorded using a LabRAM HR Evolution Raman microscope equipped with a 785 nm diode laser. Powder X-ray diffraction (XRD) patterns were collected using a Miniflex600 diffractometer with Ni-filtered Cu Kα radiation (λ = 1.542 Å) operating at 30 kV and 10 mA. The pH evolution during calcite crystallization in the presence of S_56_-B_500_ spheres and M_54_-B_200_ vesicles was monitored using a pH meter (PHS-3C, Shanghai Puchun).

**Supplementary Table 1. Synthetic recipes and GPC data for various macro-CTAs.**

**Supplementary Table 2. Synthetic recipes and GPC data for various nanoparticles.**

**Supplementary Table 3. Synthetic recipes for various nanoparticles using St and NMI as the core-forming comonomers.**

**Supplementary Fig. 1. ^1^H NMR spectra recorded for the S_56_ and M_54_ macro-CTAs.** The mean degree of polymerization (DP) was determined by end-group analysis using ^1^H NMR spectroscopy. The integrated aromatic proton signal assigned to the dithiobenzoate end-group at 7.2–8.0 ppm was compared to that of the methacrylic backbone signals at 0.5–2.5 ppm. ^1^H NMR spectra were recorded on a Bruker Avance 400 MHz spectrometer at 25 °C, using D_2_O or CD_3_OD as solvents.

**Supplementary Fig. 2. Characterization of diblock copolymer nano-objects.** Particle size distributions of **a**, S_56_-B_500_ spheres and **b**, M_54_-B_200_ vesicles, obtained from TEM images by analyzing more than 200 nanoparticles using Image J software.

**Supplementary Fig. 3. Characterization of calcite crystals precipitated in the presence or absence of anionic diblock copolymer nanoparticles. a-d,** Calcite crystals precipitated in the absence of any additives. **e-h,** Composite crystals precipitated in the presence of 0.05% w/w S_56_-B_500_ spheres. **i-l,** Composite crystals precipitated in the presence of 0.05% w/w M_54_-B_200_ vesicles. **m-p,** Composite crystals precipitated in the presence of both 0.05% w/w S_56_-B_500_ spheres and M_54_-B_200_ vesicles.

**Supplementary Fig. 4.** **Powder XRD patterns recorded for pure calcite and CaCO_3_ crystals grown in the presence of various polymeric nanoparticles.** Based on these XRD patterns, it can be concluded that the occlusion of S_56_-B_500_ spheres, M_54_-B_200_ vesicles, or both did not alter the form of the host crystal, with all composite crystals exhibiting a characteristic calcite crystallography.

**Supplementary Fig. 5. Raman spectra recorded for anionic diblock copolymer nanoparticles alone, pure CaCO_3_, and CaCO_3_ crystals precipitated in the presence of various anionic diblock copolymer nanoparticles.** Characteristic vibrational bands assigned to calcite and the nanoparticle–calcite composites are observed. In addition, the symmetric ring-breathing and in-plane C−H bending modes of the aromatic rings in the core-forming poly(benzyl methacrylate) block are detected within the composite crystals^3,4^.

**Supplementary Fig. 6. Colloidal stability of diblock copolymer nanoparticles in the presence of calcium ions at pH ~ 9. a,** Photographs of S_56_-B_500_ spheres dispersed in a series of aqueous solutions containing varying Ca^2+^ concentrations; **b,** Photographs of M_54_-B_200_ vesicles dispersed in a series of aqueous solutions containing varying Ca^2+^ concentrations; **c,** z-average hydrodynamic diameter as a function of Ca^2+^ concentration as determined by DLS; **d,** Zeta potential as a function of Ca^2+^ concentration. The error bars present the standard deviations calculated from three measurements (n = 3). It should be emphasized that the AFM force measurements and zeta potential measurements were conducted under different experimental conditions and therefore should not be directly correlated as a function of Ca^2+^ concentration.

[N.B. The S_56_-B_500_ spheres exhibited excellent colloidal stability for Ca^2+^ concentrations ranging from zero up to 5.0 mM (Supplementary Fig. 6a). In contrast, the M_54_-B_200_ vesicles began to aggregate when the Ca^2+^ concentration exceeded 3 mM (Supplementary Fig. 6b). This observation was confirmed by DLS measurements, which showed a significant increase in z-average diameter for the M_54_-B_200_ vesicles at Ca^2+^ concentrations above 3.0 mM (Supplementary Fig. 6c). In contrast, the z-average diameter of S_56_-B_500_ spheres remained constant across the 0–5.0 mM Ca^2+^ range, indicating good colloidal stability. Both S_56_-B_500_ spheres and M_54_-B_200_ vesicles interact with calcium ions, as evidenced by the significant reduction in zeta potential on addition of Ca^2+^].

**Supplementary Fig. 7. AFM-based force spectroscopy measurements of the interaction between the steric stabilizer chains (either S_56_ or M_54_) and the calcite surface.** The modified AFM tip functionalized with (a-b) S_56_ or (d-e) M_54_ chains was forced to repeatedly advance towards and retreat from the calcite surface to obtain a series of representative force-distance curves. (c, f) Force spectroscopy data indicate an increase in rupture force with increasing loading rate; the dashed lines indicate the equilibrium force (*f_eq_*) required to detach the S_56_ or M_54_ chains from the calcite surface.

**Supplementary Fig. 8. Characterization of S_30_-B_300_ vesicles and calcite crystals grown in the presence of 0.05 wt% S_30_-B_300_ diblock copolymer vesicles.** **a,** Representative TEM image recorded for S_30_-B_300_ vesicles; **b,** Z-average hydrodynamic diameter and zeta potential of S_30_-B_300_ vesicles as a function of pH as determined by DLS; **c,** Z-average hydrodynamic diameter and zeta potential of S_30_-B_300_ vesicles as a function of Ca^2+^ concentration. SEM images recorded for **d,** S_30_-B_300_@calcite composite crystals, **e,** an individual S_30_-B_300_@calcite composite crystal and **f,** a randomly fractured composite crystal. The latter image reveals that the S_30_-B_300_ vesicles are predominantly occluded within the central region of the calcite host. The error bars in **b** and **c** present the standard deviations from three independent measurements (n = 3).

**Supplementary Fig. 9. Characterization of diblock copolymer nanoparticle vesicles and calcite crystals grown in the presence of 0.05 wt% M_36_-B_200_ or M_85_-B_300_ vesicles.** **a,** TEM image and **b,** particle size distribution of M_36_-B_200_ vesicles; **c,** TEM image and **d,** particle size distribution of M_85_-B_300_ vesicles. **e-g,** Representative SEM image recorded for M_36_-B_200_@calcite composite crystals, illustrating (e) surface morphology and (f-g) internal structures; **h-j,** Representative SEM image recorded for M_85_-B_300_@calcite composite crystals, depicting (h) surface morphology and (i-j) internal structures.

**Supplementary Fig. 10. Calcite crystals precipitated in the presence of (a–d) S_56_-B_500_ spheres or (e–h) M_54_-B_200_ vesicles at [Ca^2+^] = 0.5 mM.** (**a–b, e–f**) SEM images recorded for intact composite crystals, with the insets in (**b**) and (**f**) corresponding CLSM images. (**c–d, g–h**) Representative SEM images recorded for the internal structures of randomly fractured composite calcite crystals.

[**N.B.** These composites were precipitated at an initial Ca^2+^ concentration of 0.5 mM, which is much lower than the 5.0 mM Ca^2+^ concentration used under standard conditions. Even at this relatively low Ca^2+^ concentration, which is comparable to that found in the latter stages of crystallization when starting at 5.0 mM, M_54_-B_200_ vesicles can be incorporated into the host crystals, whereas S_56_-B_500_ spheres are not occluded. This behavior is consistent with the AFM-based force spectroscopy experiments, which confirm that carboxylate groups interact more strongly with calcite crystals than sulfate groups].

**Supplementary Fig. 11. Composite crystals grown in the coexistence of two types of copolymer nanoparticles.** **a,** Representative SEM image showing the spatial distribution of M_36_-B_200_ vesicles and S_56_-B_500_ spheres within the calcite crystal. **b,** Representative SEM image showing the spatial distribution of M_54_-B_200_ vesicles and S_56_-B_500_ spheres within the calcite crystal. **c,** Representative SEM image showing the spatial distribution of M_85_-B_300_ vesicles and S_56_-B_500_ spheres within the calcite crystal. [**N.B.** To form diblock copolymer vesicles when using relatively long steric stabilizer chains, a longer core-forming block is required, which is why M_85_-B_300_ vesicles were synthesized].

**Supplementary Fig. 12. Synthesis and characterization of SK_57_-P(St-*alt*-NMI)_100_ and M_75_-P(St-*alt*-NMI)_100_ spheres prepared via RAFT-mediated PISA. a,** Synthetic protocol for SK_57_-P(St-*alt*-NMI)_100_ spheres; **b,** Synthetic protocol for M_75_-P(St-*alt*-NMI)_100_ spheres. **c,** TEM image recorded for SK_57_-P(St-*alt*-NMI)_100_ spheres; **d,** Particle size distribution of SK_57_-P(St-*alt*-NMI)_100_ spheres determined from TEM analysis; **e,** TEM image recorded for M_75_-P(St-*alt*-NMI)_100_ spheres; **f,** Particle size distribution of M_75_-P(St-*alt*-NMI)_100_ spheres determined from TEM analysis.

[N.B. For these syntheses, the poly(ammonium 2-sulfatoethyl methacrylate) steric stabilizer block was replaced with poly(3-sulfopropyl methacrylate potassium), and the degree of polymerization of the poly(methacrylic acid) steric stabilizer block was increased to 75. The core-forming block was formed by alternating copolymerization of styrene with *N*-phenylmaleimide.

**Supplementary Fig. 13. Preparation and [Ca^2+^]-dependent colloidal stability of composite nanoparticles (CNPs) by surface decoration of spherical MSNs-NH_2_ and octahedral UiO-66-NH_2_ with SK_57_-P(St-*alt*-NMI)_100_ and M_75_-P(St-*alt*-NMI)_100_, respectively. a,** Synthesis and characterization of MSNs-NH_2_/SK_57_-P(St-*alt*-NMI)_100_ CNPs; **b,** Synthesis and characterization of Uio-66-NH_2_/M_55_-P(St-*alt*-NMI)_100_ CNPs; **c,** Hydrodynamic diameters as a function of Ca^2+^ concentration as determined by DLS; **d,** Zeta potential data as a function of Ca^2+^ concentration. The error bars in **c** and **d** present the standard deviations calculated three measurements (n = 3).

Both MSNs-NH_2_ and UiO-66-NH_2_ are cationic, so electrostatic interactions enable the formation of MSNs-NH_2_/SK_57_-P(St-*alt*-NMI)_100_ and UiO-66-NH_2_/M_75_-P(St-*alt*-NMI)_100_ via surface decoration. Moreover, the distinct morphologies of spherical MSNs-NH_2_ and octahedral UiO-66-NH_2_ facilitate SEM analysis to examine their spatial distribution within host crystals.

**Supplementary Fig. 14. Calcite crystals precipitated in the presence of (a-d) MSNs-NH_2_ and (e-h) UiO-66-NH_2_ at [Ca^2+^] = 5 mM.** (**a–b, e–f**) SEM images recorded for intact crystals. (**c–d, g–h**) Representative SEM images recorded for the internal structures of randomly fractured calcite crystals, confirming that there was no occlusion in both cases.

**Supplementary Fig. 15. Composite crystals precipitated in the presence of MSNs-NH_2_/SK_57_-P(St-*alt*-NMI)_100_, UiO-66-NH_2_/M_75_-P(St-*alt*-NMI)_100_, or both at [Ca^2+^] = 5 mM. a–d**, Representative SEM images recorded for MSNs–NH_2_/SK_57_-P(St-alt-NMI)_100_ occluded within the central region of calcite crystals. **e–h**, Representative SEM images recorded for UiO-66–NH_2_/M_75_-P(St-alt-NMI)_100_ confined to the crystal surface. **i–j**, Representative SEM images illustrating the self-sorting occlusion for both types of composite nanoparticles within calcite.

**Supplementary Fig. 16. Monitoring the sequential release of vesicles and spheres.** (**a–e**) Confocal laser scanning microscopy (CLSM) images of the composite crystals acquired at different time points. (**f–j**) Corresponding scanning electron microscopy (SEM) images showing the evolution of the composite crystals over time. (**k–o**) Corresponding higher-magnification SEM images of (f–j), respectively, revealing detailed surface morphologies. White arrows indicate vesicles, whereas red arrows indicate spheres.

At *t* = 0, CLSM image showed that the spheres were embedded within the interior of the composite crystal, whereas the vesicles were distributed near the crystal surface. This spatial distribution was corroborated by SEM observations, in which vesicles were clearly identified as being anchored on the surface of the composite crystal (panel k). Upon exposure to a mildly acidic environment (pH = 4), gradual etching of calcite led to the progressive release of surface-localized vesicles. At this stage, a large number of vesicles were observed on the surface of the composite crystal as well as on the adjacent glass substrate (panel g). With further progression of the etching process, spheres began to appear on the glass substrate, indicating the release of spheres originally occluded within the interior of the composite crystal (red arrows in panels n–o). It should be noted that at pH = 4, vesicles bearing surface carboxylic acid groups undergo protonation, which induces vesicle aggregation. This accounts for the clustered vesicle assemblies observed both on the composite crystal surface and on the nearby glass substrate (panels g–i). At *t* = 90 min, even after complete dissolution of the composite crystal, a ring-like aggregation pattern resulting from vesicle clustering remained visible (dashed parallelogram). This dashed parallelogram corresponded precisely to the red rectangular region identified in the CLSM image (panel e).

**References**

1.	Chi, J., Jia, C., Zhang, W., Putnis, C. V. & Wang, L. In situ observations of the occlusion of a clay-sugar compound within calcite. *Environ. Sci: Nano* **9**, 523-531 (2022).

2.	Teng, H. H., Dove, P. M., Orme, C. A. & De Yoreo, J. J. Thermodynamics of Calcite Growth: Baseline for Understanding Biomineral Formation. *Science* **282**, 724-727 (1998).

3.	Gabrielli, C., Jaouhari, R., Joiret, S. & Maurin, G. In situ Raman spectroscopy applied to electrochemical scaling. Determination of the structure of vaterite. *J. Raman Spectrosc.* **31**, 497-501 (2000).

4.	Wehrmeister, U., Soldati, A. L., Jacob, D., Häger, T. & Hofmeister, W. Raman spectroscopy of synthetic, geological and biological vaterite: a Raman spectroscopic study. *J. Raman Spectrosc.* **41**, 193-201 (2010).
